# The Effect of Probiotic Supplementation on Cytokine Modulation in Athletes After a Bout of Exercise: A Systematic Review and Meta-Analysis

**DOI:** 10.1186/s40798-025-00860-7

**Published:** 2025-05-22

**Authors:** Diego Aparicio-Pascual, Vicente Javier Clemente-Suárez, José Francisco Tornero-Aguilera, Alejandro Rubio-Zarapuz

**Affiliations:** 1 Department of Sports Sciences. Faculty of Medicine, Health and Sports, Universidad Europea de Madrid, 28670 Villaviciosa de Odón, Madrid, Spain; 2 Grupo de Investigación en Cultura, Educación y Sociedad, Universidad de La Costa, 080002 Barranquilla, Colombia

**Keywords:** Probiotic supplementation, Cytokine modulation, Exercise-induced inflammation, Athletes, Systematic review, Meta-analysis

## Abstract

**Background:**

Exercise-induced inflammation, especially after intense or prolonged physical activity, can hinder recovery in athletes. Probiotic supplementation has been suggested as a potential method to modulate this inflammatory response by influencing the gut microbiota. However, the effects of probiotics on cytokine profiles following exercise remain unclear. This systematic review and meta-analysis aimed to assess the impact of probiotic supplementation on cytokine modulation in athletes aged 18–50 years following exercise.

**Methods:**

Randomized controlled trials (RCTs) that administered probiotic supplementation for at least one week to athletes were included. Studies comparing probiotics to a placebo or no supplementation, with post-exercise cytokine levels as the primary outcome, were analyzed. A systematic search was conducted across four databases (PubMed (Medline), Scopus, Web of Science (WOS) and Cochrane), up to June 2024. Risk of bias was assessed using the McMaster Critical Review Form, and random-effects meta-analyses were performed to determine the impact of probiotic supplementation.

**Results:**

A total of 19 studies involving 526 athletes from various endurance disciplines were included in the review. Probiotic supplementation significantly increased the anti-inflammatory cytokine interleukin-10 (IL-10) (SMD = 0.43; 95% CI 0.25–0.61; I^2^ = 0%). However, no significant effects were observed for other cytokines, including IL-1β, IL-6, IL-8, TNF-α, or IFN-γ. Subgroup analyses supported the consistency of IL-10 findings across different exercise protocols, though substantial heterogeneity was observed for some cytokines. The variability in study designs, probiotic strains, dosages, and exercise modalities contributed to the mixed results.

**Conclusion:**

Probiotic supplementation appears to enhance anti-inflammatory responses post-exercise, particularly by increasing IL-10 levels, which may aid recovery in athletes. However, the evidence regarding its effects on pro-inflammatory cytokines remains inconclusive. Further well-designed RCTs are needed to clarify these effects and establish standardized protocols for supplementation.

**Supplementary Information:**

The online version contains supplementary material available at 10.1186/s40798-025-00860-7.

## Key Points

- Probiotic supplementation enhances anti-inflammatory responses post-exercise, particularly by increasing IL-10 levels, suggesting a potential benefit for athletes in terms of recovery. This effect may be mediated through modulation of the intestinal microbiota, which plays a critical role in immune regulation.
- Despite the observed increase in IL-10, probiotics did not significantly alter the levels of pro-inflammatory cytokines such as IL-1β, IL-6, IL-8, TNF-α, or IFN-γ. This indicates that the immunomodulatory effects of probiotics may be selective, primarily enhancing anti-inflammatory responses rather than suppressing inflammation directly.
- Differences in probiotic strains, dosages, exercise modalities, and study designs contributed to variability in results. Future research should aim to establish standardized supplementation protocols to clarify the role of probiotics in cytokine modulation and athletic recovery.

**Supplementary Information:**

The online version contains supplementary material available at 10.1186/s40798-025-00860-7.

## Background

Probiotic supplementation has garnered significant attention in the realm of sports nutrition, particularly due to its potential role in modulating immune responses following exercise. They are defined as living microorganisms that can confer health benefits to the host when administered in adequate amounts [1, 2]. These living microorganisms typically consist of several strains, with the most significant being bacteria from the genera *Lactobacillus* and *Bifidobacterium*, as well as yeasts from the genus *Saccharomyces*. It is important to note that probiotic supplements can be composed of either a single strain or multiple strains [3]. Currently, they are used to alleviate GI problems and overtraining issues which appear frequently in endurance sports [4]. Additionally, they appear to improve nutrient absorption and digestion capacity [5]. Their mechanisms of action include enhancing intestinal barrier function, regulating the production of antimicrobial peptides and antioxidant compounds/enzymes, involving SCFAs in balancing regulatory T lymphocytes and modifying cytokine secretion by macrophages and lymphocytes [2, 5]. However, these mechanisms are profoundly affected by the composition of the gut microbiota, which notably influences how probiotics exert their effects [6].

A central component of immune regulation, particularly in response to exercise, involves cytokines, small signaling proteins that facilitate communication between immune cells. Cytokines coordinate the body’s inflammatory and anti-inflammatory responses, ensuring a proper balance between necessary immune activation and the resolution of inflammation. For readers less familiar with these biomarkers, cytokines are typically classified into pro-inflammatory and anti-inflammatory categories, each with distinct roles in immune regulation [7, 8].

Pro-inflammatory cytokines, are primarily responsible for initiating the inflammatory response, mobilizing immune cells to sites of tissue damage, and promoting repair mechanisms. such as interleukin-6 (IL-6) and tumor necrosis factor-alpha (TNF-α), are essential in the initial response to infection, injury, or exercise. These cytokines serve to mobilize immune cells to sites of tissue damage and promote repair mechanisms essential for recovery. IL-6, produced by contracting muscles during exercise, is known to stimulate acute-phase protein production and influence immune cell differentiation, facilitating the body’s initial response to stress. TNF-α, a potent mediator of systemic inflammation, can induce fever, trigger apoptosis in damaged cells, and stimulate the production of additional pro-inflammatory cytokines. This cascade of responses ensures the repair and adaptation processes are activated but can contribute to muscle soreness and delayed recovery when elevated excessively or for prolonged periods [7]. Anti-inflammatory cytokines, particularly interleukin-10 (IL-10), counterbalance the effects of pro-inflammatory cytokines, promoting the resolution of inflammation once the initial threat has been addressed. This balance between pro-inflammatory and anti-inflammatory cytokines is crucial for maintaining immune homeostasis and preventing chronic or excessive inflammation. IL-10 plays a central role in deactivating immune responses that could potentially harm healthy tissues. The interplay between these cytokine types is particularly relevant during and after physical exercise, where the body experiences a transient inflammatory response that is essential for the normal physiological adaptation process. This response is characterized by an increase in pro-inflammatory cytokines, which help initiate tissue repair and facilitate adaptation to physical stressors [7]. This balance is particularly crucial during and after physical exercise, when the body experiences a transient inflammatory response essential for physiological adaptation. Pro-inflammatory cytokines increase during exercise, initiating repair processes and supporting adaptations to physical stress. Understanding cytokine dynamics is key to developing effective recovery strategies for athletes and active individuals. Elevated levels of these cytokines reflect the body’s natural response to stress, playing a dual role in healing and adaptation. However, managing this response can be challenging, especially when targeting cytokine modulation [8]. Table 1 summarizes the key roles of cytokines in relation to exercise, highlighting their involvement in the pro or anti- inflammatory response and physiological adaptations.

**Table 1:** Summary of the roles of key cytokines in relation to exercise

Cytokine	Actions	Change with exercise
“Pro-inflammatory”		
IL-1β	Induce synthesis of NO, prostaglandins and leukotrienes	↑ ↔
IL-8	Neutrophils chemotaxis Induction of angiogenesis	↑
IL-15	Activation of adaptative immune system (B cells and T cells)	=
TNF-α	Immune cell activation Stimulation of prostaglandins synthesis	↑
“Anti-inflammatory”		
IL-1Ra	Inhibition of signaling through IL-1 receptor	↑
IL-4 & IL-13	Inhibition of Th1 cells Reduction of plasma IL-1β Upregulation of IL-1Ra expression	↑ ↔
IL-6	Induces upregulation of IL-10 and Il-1Ra “Pro-inflammatory” cytokine inhibition	↑
IL-10	Inhibition of “pro-inflammatory” cytokines including IL-1β and TNF-α	↑

Adapted from [7]. NO: Nitric oxide; IL-1β: Interleukin 1-β; IL-8: Interleukin 8; IL-15: Interleukin 15; TNF-α: Tumor necrosis factor α; IL-1Ra: Interleukin 1-Ra; IL-4: Interleukin 4; IL-13: Interleukin 13; Th1 cells: T helper 1 cells; IL-6: Interleukin 6; IL-10: Interleukin 10; ↑: upregulated after exercise; = : no change with exercise; ↑ ↔ : inconsistent findings

In this context, sports nutrition offers various approaches to support recovery, reduce injury risk, and enhance overall performance. As abovementioned probiotic supplementation, has emerged as a promising strategy, some meta-analyses suggest that probiotics not only modulate inflammation but also contribute to aerobic performance improvements in athletes, presenting an innovative tool for optimizing training and recovery outcomes [5, 9, 10]. Related with these roles, they are widely recognized for their role in reducing the incidence of upper respiratory tract infections (URTIs) by enhancing immune responses through T- and B-lymphocyte activation and dampening intestinal inflammation. This immunomodulatory effect promotes the secretion of key cytokines, including interferon-gamma (IFN-γ), immunoglobulin A (IgA), and IL-10, while concurrently downregulating pro-inflammatory cytokines such as IL-6, IL-8, and TNF-α. [1, 5, 11]. As highlighted by Jäger et al. [1], achieving these potential benefits require a minimum dosage of 1 × 10⁹ colony-forming units (CFU), during at least 3 weeks for most bacterial species. Despite research highlighting the benefits of probiotics in reducing URTIs and gastrointestinal symptoms, studies examining their effects on cytokine modulation in athletes remain limited. Only two meta-analyses have specifically examined probiotics' effects on cytokine responses in this population, each encompassing fewer than ten studies, which constrains the robustness of the conclusions drawn [9, 10]. While these reviews have identified positive outcomes for certain cytokines, the findings remain inconsistent, underscoring the need for a comprehensive meta-analysis that synthesizes current evidence on cytokine modulation following exercise. Consequently, a comprehensive meta-analysis is crucial to assess the effectiveness of probiotics in modulating cytokine responses following exercise. The current literature is limited and inconsistent, undermining the reliability of existing conclusions. This meta-analysis seeks to synthesize available data to enhance our understanding of probiotics' role in improving immune function and recovery in athletes.

This meta-analysis evaluates the effects of probiotic supplementation on cytokine modulation in athlete’s post-exercise. By synthesizing existing research, it aims to clarify the role of probiotics in managing inflammation and supporting recovery.

## Methods

Following the guidelines of the PRISMA® statement (Preferred Reporting Items for Systematic Reviews and Meta-Analyses) [12], a systematic review of the scientific literature was conducted to examine the impact of probiotic supplementation on cytokine modulation after a bout of exercise in different types of athletes.

### Search Strategy

Records were identified through extensive searches of the scientific databases PubMed (Medline), Scopus, Web of Science (WOS) and Cochrane on June 28th, 2024. The key terms, along with the Boolean algorithms and wildcards used in the search string, were as follows:

- PubMed (Medline): ("probiotic*" [TIAB] OR "probiotic*" [TW] OR "microbiota*" [TIAB] OR "microbiota*" [TW] OR "postbiotic*" [TIAB] OR "postbiotic*" [TW]) AND ("cytokine?" [TIAB] OR "cytokine?" [TW] OR "inflamm*" [TIAB] OR "inflamm*" [TW] OR "immune*" [TIAB] OR "immune*" [TW]) AND ("exercise" [TIAB] OR "exercise" [TW] OR "athletes" [TIAB] OR "athletes" [TW] OR "endurance athletes" [TIAB] OR "endurance athletes" [TW])
- Scopus, Web of Science (WOS) and Cochrane: ("probiotic*" OR "microbiota*" OR "postbiotic*") AND ("cytokine?" OR "inflamm*" OR "inmune*") AND ("exercise" OR "athletes" OR "endurance athletes")

**Fig. 1:**
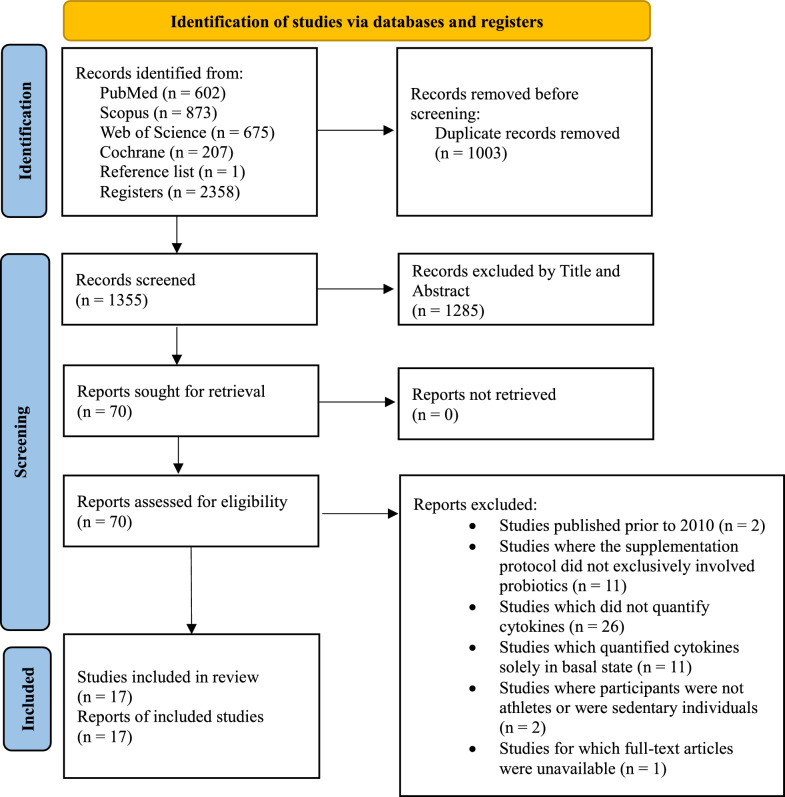
PRISMA 2020 flow diagram for new systematic reviews which included searches of databases and registers only

Only English-language peer-reviewed scientific articles published from 2010 to June 28, 2024, were included, independently of risk of bias assessment scores. Grey literature, such as government reports and unpublished theses, were not specifically screened. All steps in the record screening process were managed using Zotero® version 6.0. Additionally, references from the included records were manually searched for potential additional sources. Therefore, screening process is summarized in Fig. 1.

### Eligibility Criteria

A total of 2358 records were identified across the four databases used. Additionally, a review of the reference lists from the articles revealed one additional record, which was included in the final review. The selection process was conducted by two authors (DAP and ARZ), with a third author (VJCS) consulted to resolve any discrepancies and provide the final decision. Duplicate entries were manually removed, resulting in 1355 records for further screening. After eliminating 1285 records based on title and abstract, 70 records were assessed for full text. Ultimately, 17 records were included, with one being obtained through citation searches.

Out of these, 17 records were included in the qualitative and quantitative synthesis (meta-analysis), while the analyses encompassed a total of 19 articles. The discrepancies occurred because the study by Huang et al. [13] involved two distinct populations, and the study by West et al. [14] presented results separated by gender. Consequently, a specific citation approach was adopted for each group. Participants from the first intervention were referenced as "1" (Huang et al. [13]), while those from the second intervention were labeled as "2" (Huang et al. [13]). A similar strategy was employed to differentiate between female and male participants in the West et al., 2011 study, with females cited as (West et al. [14]) and males as (West et al. [14]). This citation strategy ensured a clear distinction between the populations within the meta-analysis framework, facilitating the interpretation of the findings.

The eligibility criteria for record screening were established based on the following PICOS framework:

- Participants: athletes, ranging from 18 to 50 years of age.
- Intervention: Probiotic supplementation for at least one week.
- Comparator: age-matched athletes.
- Outcomes: post exercise circulating levels of cytokines, acute phase proteins, tumor necrosis factor α (TNF-α), interferon-ƴ (IFN-ƴ) and C-reactive protein (CRP).
- Study design: Randomized trials reporting inflammatory marker values measured after a bout of exercise following a probiotic or placebo intervention.

Prior to full-text screening, all systematic and narrative reviews were excluded based on title and abstract. Exclusion criteria for articles not included in the meta-analysis were as follows: studies published before 2010 were excluded, along with those where the supplementation protocol included did not exclusively involved probiotics. Additionally, research was excluded if it did not measure cytokine levels or only assessed them at baseline. Studies involving non-athletes or sedentary individuals were also not considered. Finally, any studies without accessible full-text articles were excluded from the review.

### Risk of Bias Assessment

**Fig. 2:**
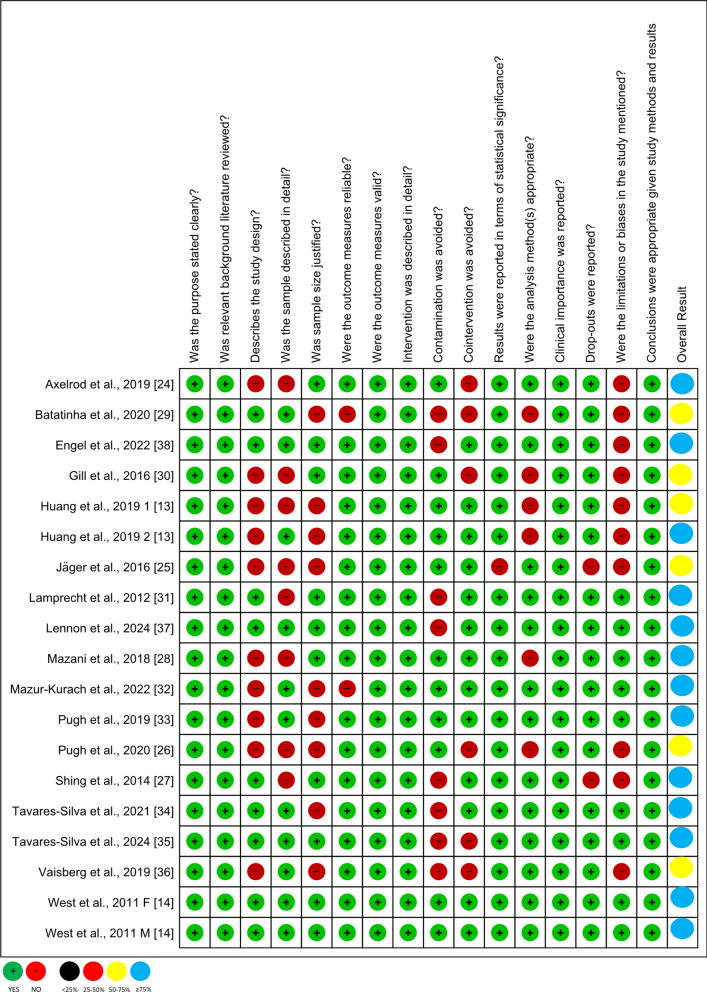
Summary of bias assessment with mcmaster scale

The risk of bias in the included studies was evaluated using the Critical Review Form-Quantitative Studies, also known as the McMaster Scale [15], as shown in Fig. 2. The lead author (DAP) conducted the initial risk of bias assessment, which was then reviewed for accuracy by all co-authors. This evaluation incorporated 16 criteria instead of the standard 15, with an additional criterion specifically addressing study limitations. Special attention was given to the randomization process: studies that did not report the randomization procedure, allocation process, or provide a CONSORT diagram were marked as "no" for that criterion. Based on the critical appraisal results, an overall methodological quality score was assigned: studies were classified as having "very low" quality, indicated by black, if fewer than 25% of criteria were met; "low" quality, indicated by red, if 25–50% of criteria were met; "moderate" quality, indicated by yellow, if 50–75% of criteria were met; and "high" quality, indicated by blue, if 75% or more of the criteria were met.

### Data Extraction

Data extraction was conducted by the lead author (DAP) and independently reviewed by (ARZ) and (JFTA) to ensure accuracy and reliability. To facilitate comparisons between study groups, effect measures for continuous outcomes were collected, including mean and standard deviation (mean ± SD), mean and standard error (mean ± SE), median and interquartile range (median [IQR]), and log-transformed values of circulating inflammatory markers at baseline. Prior to analysis, data using the formula SD = SE x $\sqrt{n}$, in which n is the sample size for each group. Mean ± SD values were also estimated from median (IQR) data using the method proposed by Wan et al. [16]. It is important to note that in the study by West et al. [14], data is reported as log-transformed values. However, conversion of this data was not necessary, as the supplementary material of the study provides the raw data. Also, estimated values from graphical representations (bar, line chart…) were extracted using WebPlotDigitizer® version 4.8.

Once extracted, these values were added to a template in Microsoft Excel®, which included the following fields: citation, supplementation period, participants, participants divided by the supplementation groups (probiotic/placebo), method of data extraction, reported values (pg/ml), data reported values ((Mean ± SD), (Mean ± SE) and (Median ± Interquartile range)), bout of exercise and the data of the cytokines already transformed to (Mean ± SD).

### Statistical Analysis

The analysis was conducted using Jamovi® version 2.6.13, utilizing the standardized mean difference as the outcome measure and applying a random-effects model to fit the data. The heterogeneity (tau^2^) was estimated using Hedges' estimator [17]. Alongside the tau^2^ estimate, the Q-test for heterogeneity [18] and the I^2^ statistic were reported. When any heterogeneity was detected (tau^2^ > 0), regardless of the Q-test results, a prediction interval for the true outcomes was provided. To identify potential outliers and influential studies within the model, studentized residuals and Cook's distances were examined. Studies with studentized residuals exceeding the 100 x (1—0.05/(2 k))^th^ percentile of a standard normal distribution were flagged as potential outliers, applying a Bonferroni correction with a two-sided alpha of 0.05 for k included studies. Studies were considered influential if their Cook's distance was greater than the median plus six times the interquartile range of all Cook's distances. Funnel plot asymmetry was assessed using both the rank correlation test and the regression test, with the standard error of the observed outcomes serving as the predictor. However, this was only assessed when a minimum of 10 studies were available, this was done according to the guidelines implemented by Godavitarne et al. [19]. In which, due to the low formal power of the analysis, it is unable to distinguish between chance and true asymmetry. In other words, despite ruling out asymmetry, bias cannot be excluded. Although Jamovi reported heterogeneity with three different measurements tau^2^, Q and I^2^ values heterogeneity in this study it was opted for I^2^ for heterogeneity assessment. Which is a reliable, scale-independent measure in meta-analysis that effectively quantifies variability arising from differences among studies [20]. However, it may be biased when fewer than seven studies are included in the analysis [21]. As so, to address this potential bias, the analysis was only conducted when at least seven distinct studies reported on the same cytokine, following the previously mentioned criteria. Therefore, heterogeneity is considered low for values less than 30%, moderate for values greater than 30%, substantial for values greater than 50%, and considerable for values greater than 75% [22]. Finally, regarding subgroup analyses they were conducted to account for overly influential studies, when they were identified through studentized residuals and Cook’s distances. Furthermore, the studies were categorized based on exercise bout, specifically differentiating between VO2max tests and marathon/triathlon events and if applicable overly influential studies were again eliminated. But it is important to note that these analyses were performed only when a minimum of seven studies as it is mentioned previously.

A multivariate random-effects meta-regression was conducted to evaluate whether specific covariates, number of probiotic strains, exercise type, and intervention duration, could predict the effects of probiotics on cytokine modulation in athletes. The meta-regression analysis was performed using Open Meta-Analyst software® when heterogeneity exceeded 75% and at least 15 studies were available, following the recommendations of Thompson and Higgins [23].

The covariates were categorized as follows: the probiotic strains were classified based on the specific number of strains used in each study protocol, the intervention duration was categorized according to the number of weeks each study lasted, and the type of exercise was assigned a binary classification, studies involving prolonged endurance activities such as marathons and triathlons were coded as 0, whereas those incorporating VO₂ max tests were coded as 1. These categorizations allowed for a structured analysis of how different probiotic interventions and exercise modalities influence cytokine responses in athletes.

## Results

A total of 526 athletes were documented in the included studies, with 240 participating in probiotic supplementation and 247 in placebo supplementation, in addition to 39 athletes included in various crossover designs [24–27]. These athletes were further categorized into various endurance sports disciplines (runners, marathoners, cyclists and triathletes), except for those in the study by Jäger et al. [25], which involved resistance-trained athletes, and the study by Mazani et al. [28], which did not specify the sport discipline of the athletes. The age range of the athletes spanned from a minimum of 18 to a maximum of 50 years. While most studies reported VO2max, it was not consistently documented across all investigations. Regarding sex, the studies by (West et al. [14, 25, 27, 29–36]) included only male athletes. However, the studies [33, 37], included both sexes, while the studies by (West et al. [14, 28]) included only female athletes. Finally, the sex of the athletes was not specified in the studies by (Huang et al. [13]; Huang et al. [13, 26, 38]). It is worth noting that several Lactobacillus species were reclassified in 2020 [31]; however, this study used the names reported in the original studies to minimize bias.

Additional information about this data can be found in Table 2, which details the study design, sample size, posology, bacterial species used, exercise bout conducted before the cytokine’s extraction and study outcomes (Table 3).

**Table 2:** Characteristics of included studies

Study	Design	Sample	Posology	Bacterial species	Bout of exercise	Outcomes
Axelrod et al. [24]	Double-blind randomized, placebo-controlled trial	Seven endurance trained athletes, sex not reported; aged 18–45 years; VO2max > 50 ml/kg/min	One capsule per day, for a duration of 4 weeks	*Lactobacillus salivarius UCC118* (2 × 10^8^ CFU/capsule)	Two hours of continuous aerobic exercise at 60% of VO2 max	PRO VS PLA: = IL-6
Batatinha et al. [29]	Double-blind randomized trial	Twenty-seven marathonists, males; aged 30–45 years; VO2max not reported	One sachet per day, for a duration of 30 days	*Bifidobacterium animalis. subsp. Lactis* (10 × 10^9^ CFU/sachet) and *Lactobacillus acidophilus* (10 × 10^9^ CFU/sachet)	Marathon race (42.195 km)	PRO VS PLA: = IL-1β, IL-2, IL-4, IL-6, IL-8, IL-10, IL-15, TNF-α and IFN-ƴ
Engel et al. [38]	Double-blind randomized, two-armed, placebo-controlled trial	One-hundred and twenty-six endurance athletes, sex not reported; aged 18–50 years; VO2max > 40 ml/kg/min	Two capsules per day, one with breakfast, for a duration of 6 weeks	*Bifidobacterium breve Bif195* (2.5 × 10^10^ CFU/capsule)	One hour of continuous aerobic exercise at 80% of VO2 max	PRO VS PLA: = IL-1β, IL-2, IL-4, IL-6, IL-8, IL-10, IL-12p70, IL-13, TNF-α and IFN-ƴ; ↑ CRP
Gill et al. [30]	Blinded randomized and counterbalanced order trial	Eight non-heat acclimatized healthy endurance athletes, males; aged 26 ± 6 years; VO2max 59 ± 5 ml/kg/min (Mean ± SD)	One beverage per day, for a duration of 7 days	*Lactobacillus casei* (1 × 10^11^ CFU/beverage)	Two hours of continuous aerobic exercise at 60% of VO2 max, with ambient conditions of 34 ± 0,4 °C and 32 ± 2% relative humidity	PRO VS PLA: = IL-1β, IL-6, IL-8, IL-10, TNF-α and IFN-ƴ
Huang et al. [13]	Double-blind randomized trail	Nineteen triathletes, sex not reported; aged 20.65 ± 1.1 years; VO2max not reported (Mean ± SD)	Two capsules per day, for a duration of 4 weeks	*Lactobacillus plantarum PS128* (1.5 × 10^10^ CFU/capsule)	Sprint Triathlon (750 m swimming, 20 km biking and 5 km running)	PRO VS PLA: = IL-4 and IL-10; ↓ IL-6, IL-8 and TNF-α; ↑ IFN-ƴ
Huang et al. [13]	Double-blind randomized trial	Eighteen triathletes, sex not reported; aged 21.2 ± 0.75 years; VO2max 60.95 ± 1.85 ml/kg/min (Mean ± SD)	Two capsules per day, for a duration of 3 weeks	*Lactobacillus plantarum PS128* (1.5 × 10^10^ CFU/capsule)	Official competition of Triathlon (1.5 km swimming, 40 km biking and 10 km running)	PRO VS PLA: ↓ IL-6, IL-8 and TNF-α and IFN-ƴ; ↑ IL-4 and IL-10
Jäger et al. [25]	Double-blind, randomized, placebo-controlled, crossover trial	Fifteen resistance-trained, males; aged 25 ± 4 years; VO2max not reported (Mean ± SD)	One capsule per day, for a duration of 21 days	*Bifidobacterium Breve BR03* (5 × 10^9^ CFU/capsule) and *Streptococcus thermophilus FP4* (5 × 10^9^ CFU/capsule)	5 sets of 10 maximal eccentric (forced lengthening) contractions at a speed pf 30°/s	PRO VS PLA: = IL-6
Lamprecht et al. [31]	Double-blind randomized, placebo-controlled trial	Twenty-three endurance trained, males; aged 30–45 years; VO2max > 45 mL/kg/min	Two sachets per day, with 100–125 ml of plain water for a duration of 14 weeks	*Bifidobacterium bifidum W23, Bifidobacterium lactis W51, Enterococcus faecium W54, Lactobacillus acidophilus W22, Lactobacillus brevis W63,* and *Lactococcus lactis W58*; with a minimal concentration of 2.5 × 10^9^ CFU/sachet	Test to exhaustion in a cycle ergometer work started at 60 Watts (W) for three minutes and was increased 20 W every minute until voluntary exhaustion. It lasted around 15 to 18 min	PRO VS PLA: = IL-6 and TNF-α
Lennon et al. [37]	Double-blind randomized, placebo-controlled cross-over study	Sixteen runners, males and females; 32.7 ± 8 years; VO2max not reported (Mean ± SD)	One capsule per day for a duration of 4 weeks	*Pediococcus acidilactici* and two *Lactobacillus plantarum strains* maintaining an equal ratio of 1:1:1; with a minimal concentration of 3.0 × 10^9^ CFU/capsule	90 min of test to exhaustion in a treadmill, where participants ran at 65–70% VO2max	PRO VS PLA: = IL-1β, IL-6, IL-8, IL-10 and TNF-α
Mazani et al. [28]	Blind randomized trial	Twenty-seven athletes, females; aged 18–25 years; VO2max not reported	Two cups of probiotic yoghurt (450 g) per day, for a duration of 2 weeks	Not addressed	Test to exhaustion according to Bruce test	PRO VS PLA: = IL-6 and TNF-α
Mazur-Kurach et al. [32]	Double-blind randomized trial	Twenty-six elite road cyclist, males; aged 18–26 years; VO2max 61.66 ± 3.26 ml/kg/min (Mean ± SD)	One capsule per day, for 16 weeks	*Lactobacillus plantarum*, *Lactobacillus casei*, *Lactobacillus rhamnosus*, *Bifidobacterium breve*, *Lactobacillus acidophilus*, *Bifidobacterium longum*, *Bifidobacterium bifidum*, *Bifidobacterium infantis*, *Lactobacillus helveticus*, *Lactobacillus fermentum*, *Lactobacillus bulgaricus, Lactococcus lactis*, and *Streptococcus thermophilus*; with a minimal concentration of 1 × 10^11^ CFU/capsule	Cycle ergometer, test started with a load of 100 W; then every two minutes the resistance was increased by 35 W. The pedaling rhythm was constant at 90 rpm till exhaustion	PRO VS PLA: = IL-1β, IL-6 and IL-8 ↓ TNF-α; ↑ IL-10
Pugh et al. [33]	Double-blind randomized, matched-pairs trial	Twenty runners, males and females; aged 35.45 ± 7.2 years; VO2max 57 ± 8.3 ml/kg/min (Mean ± SD)	One capsule per day, for a duration of 28 days and an extra one two hours prior to the race	*Lactobacillus acidophilus (CUL60), Lactobacillus acidophilus (CUL21), Bifidobacterium bifidum (CUL20),* and *Bifidobacterium animalis subsp. lactis* (CUL34); with a minimal concentration of 2.5 × 10^10^ CFU/capsule	Marathon race (42.195 km)	PRO VS PLA: = IL-6, IL-8 and IL-10
Pugh et al. [26]	Double-blind randomized, placebo-controlled crossover trial	Seven trained cyclists, sex not reported; aged 23 ± 4 years; VO2max 64 ± 2.2 ml/kg/min (Mean ± SD)	One capsule per day, for a duration of 28 days	*Lactobacillus acidophilus (CUL60), Lactobacillus acidophilus (CUL21), Bifidobacterium bifidum (CUL20), and Bifidobacterium animalis subsp. lactis (CUL34);* with a minimal concentration of 2.5 × 10^10^ CFU/capsule	Two hours in a cycle ergometer at 55% Wmax and a cadence dependent linear output of 100 kJ of work	PRO VS PLA: = IL-1a, IL-8 and IL-10 ↓ IL-6
Shing et al. [27]	Double-blind randomized, placebo-controlled and counterbalanced crossover trial	Ten trained runners, males; aged 27 ± 2 years; VO2max 62.6 ± 2.1 ml/kg/min (Mean ± SD)	One capsule per day, for a duration of 4 weeks	*Lactobacillus acidophilus* (7.4 × 10^9^ CFU/capsule); *L. rhamnosus* (15.55 × 10^9^ CFU/capsule); *L. casei* (9.45 × 10^9^ CFU/capsule); *L. plantarum* (3.15 × 10^9^ CFU/capsule); *L. fermentum* (1.35 × 10^9^ CFU/capsule); *Bifidobacterium lactis* (4.05 × 10^9^ CFU/capsule); *B. breve* (1.35 × 10^9^ CFU/capsule); *B. bifidum* (0.45 × 10^9^ CFU/capsule); *Streptococcus thermophilus* (2.25 × 10^9^ CFU/capsule); with a minimal concentration of 45 × 10^9^ CFU/capsule	Time to fatigue run was assessed during a treadmill run set at 80% of ventilatory threshold, conducted in a climate chamber set at 35 °C and 40% relative humidity	PRO VS PLA: = IL-1ra, IL-6, IL-10 and TNF-α
Tavares-Silva et al. [34]	Double-blind randomized trial	Fourteen marathon runners, males; aged 39.92 ± 3.47 years; VO2max 55.72 ± 7.46 ml/kg/min (Mean ± SD)	One gelatinous capsule per day during nighttime, for a duration of 30 days	*Lactobacillus acidophilus-LB-G80* (1 × 10^9^ CFU/capsule); *Lactobacillus paracasei-LPc-G110* (1 × 10^9^ CFU/capsule); *Lactococcus subp. lactis-LLL-G25* (1 × 10^9^ CFU/capsule); *Bifidobacterium animalis subp. lactis-BL-G101* (1 × 10^9^ CFU/capsule); *Bifidobacterium bifidum-BB-G90* (1 × 10^9^ CFU/capsule); with a minimal concentration of 5 × 10^9^ CFU/capsule	International São Paulo Marathon (42.195 km)	PRO VS PLA: = IL-2, IL-4, IL-10 and TNF-α
Tavares-Silva et al. [35]	Double-blind randomized, controlled trial	Twenty-seven marathoners, males; aged 25–45 years; VO2max not reported	One sachet per day, for a duration of 30 days	*Lactobacillus acidophillus* (1 × 10^10^ CFU/capsule) and *Bifidobacterium bifidum subsp. Lactis* (1 × 10^10^ CFU/capsule)	International São Paulo Marathon (42.195 km)	PRO VS PLA: = L-1β, IL-6, IL-10 and TNF-α; ↓ IL-15; ↑ IL-8
Vaisberg et al. [36]	Blind randomized trial	Forty-two amateur marathon runners, males; aged 39.5 ± 9.4 years; VO2max 57.75 ± 6.87 (Mean ± SD)	One bottle of fermented milk (80 g) per day, for a duration of 30 days	*Lactobacillus casei Shirota* (40 × 10^9^ CFU/beverage)	Marathon race (42.195 km)	PRO VS PLA: = IL-1ra, IL-1β, IL-4, IL-6, IL-10, IL-12p70 and IL-13; ↓ TNF-α
West et al. [14]	Double-blind randomized, placebo-controlled parallel-groups trial	Thirty-five cyclist and triathletes, females; aged 36.05 ± 9.4 years; VO2max > 45 ml/kg/min	One capsule per day, for a duration of 11 weeks	*Lactobacillus fermentum VRI-003* (1 × 10^9^ CFU/capsule)	Incremental performance test in a cycle ergometer	PRO VS PLA: = IL-8, IL-10 and IFN-ƴ; ↓ IL-1ra, IL-6 and TNF-α
West et al. [14]	Double-blind randomized, placebo-controlled parallel-groups trial	Sixty-two cyclist and triathletes, males; aged 35.8 ± 9.6 years; VO2max > 50 ml/kg/min (Mean ± SD)	One capsule per day, for a duration of 11 weeks	*Lactobacillus fermentum VRI-003* (1 × 10^9^ CFU/capsule)	Incremental performance test in a cycle ergometer	PRO VS PLA: = IL-6, IL-8, TNF-α and IFN-ƴ; ↓ IL-1ra and IL-10

VO2max: Maximum Oxygen Intake; CFU: Colony-Forming Units; PRO: Probiotics; PLA: Placebo; IL: Interleukin; TNF: Tumor Necrosis Factor; IFN: Interferon; CRP: C Reactive Protein; W: Watts; RPM: Revolutions Per Minute, kJ: Kilojoules; = there was no statistical significance difference; ↓: there was statistical significance difference and the concentration decreased; ↑: there was statistical significance difference and the concentration increased

**Table 3:** Summary of statistics of all cytokines

Cytokine	Number of studies (k)	Effect size (Mean, 95% CI)	*p*-value	Heterogeneity (I^2^) (%)	Q-test (Q, *p*-value)	Prediction interval	Potential outliers	Influential studies	Funnel plot asymmetry
IL-1β	7	0.065 (− 0.467 to 0.598)	0.810	73.12	27.031 (0.0001)	− 1.209 to 1.340	Batatinha et al. [29]	Batatinha et al. [29]	NA
IL-6	18	− 0.124 (− 0.331 to 0.083)	0.239	25.11	25.841 (0.077)	− 0.597 to 0.349	None	None	Yes (p = 0.028, regression test)
IL-8	12	− 0.156 (− 0.502 to 0.190)	0.376	58.98	24.346 (0.0113)	− 1.094 to 0.781	Batatinha et al. [29]	Batatinha et al. [29]	No
IL-10	15	0.434 (0.252 to 0.617)	< 0.0001	0.00	9.605 (0.791)	N/A	None	Engel et al. [38]	No
TNF-α	15	− 0.283 (− 0.733 to 0.167)	0.218	80.57	93.474 (< 0.0001)	− 1.870 to 1.304	West et al. M [14]	West et al. [14]	No
IFN- ƴ	7	0.972 (− 1.077 to 3.022)	0.352	97.65	135.650 (< 0.0001)	− 4.727 to 6.671	West et al. [14]	None	NA

NA: Not Addressed

### IL-1β

**Fig. 3:**
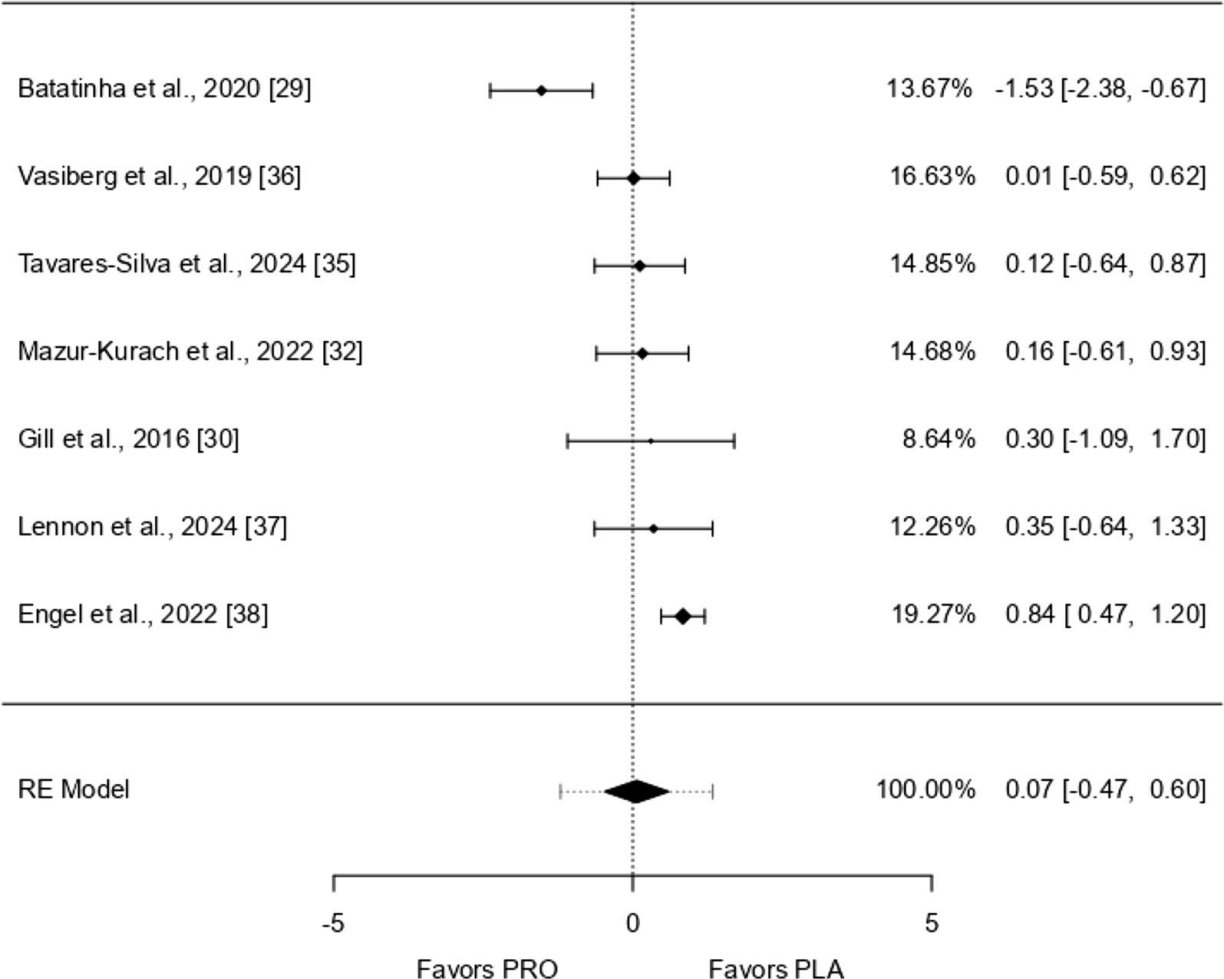
IL-1β forest plot

**Fig. 4:**
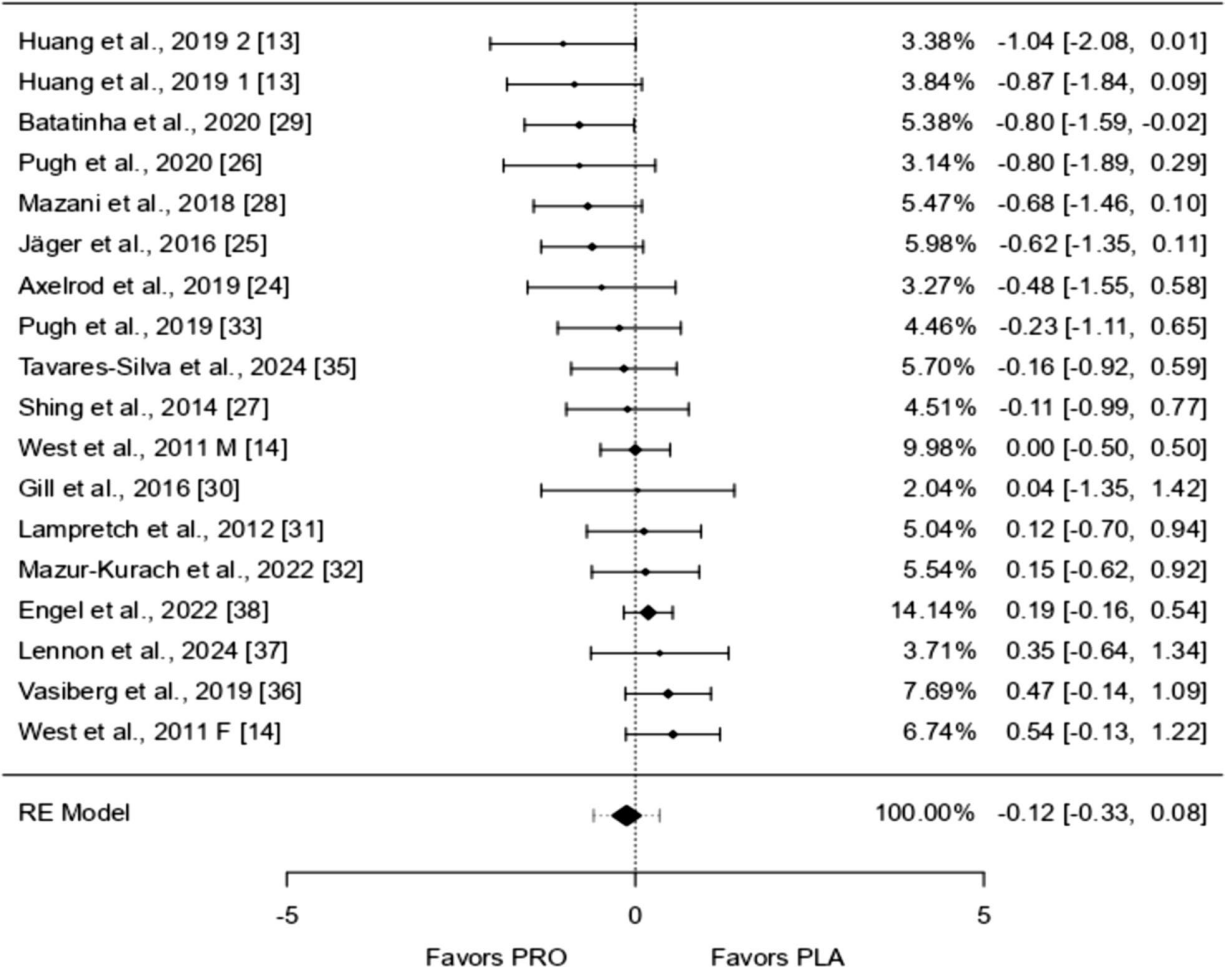
IL-6 forest plot

**Fig. 5:**
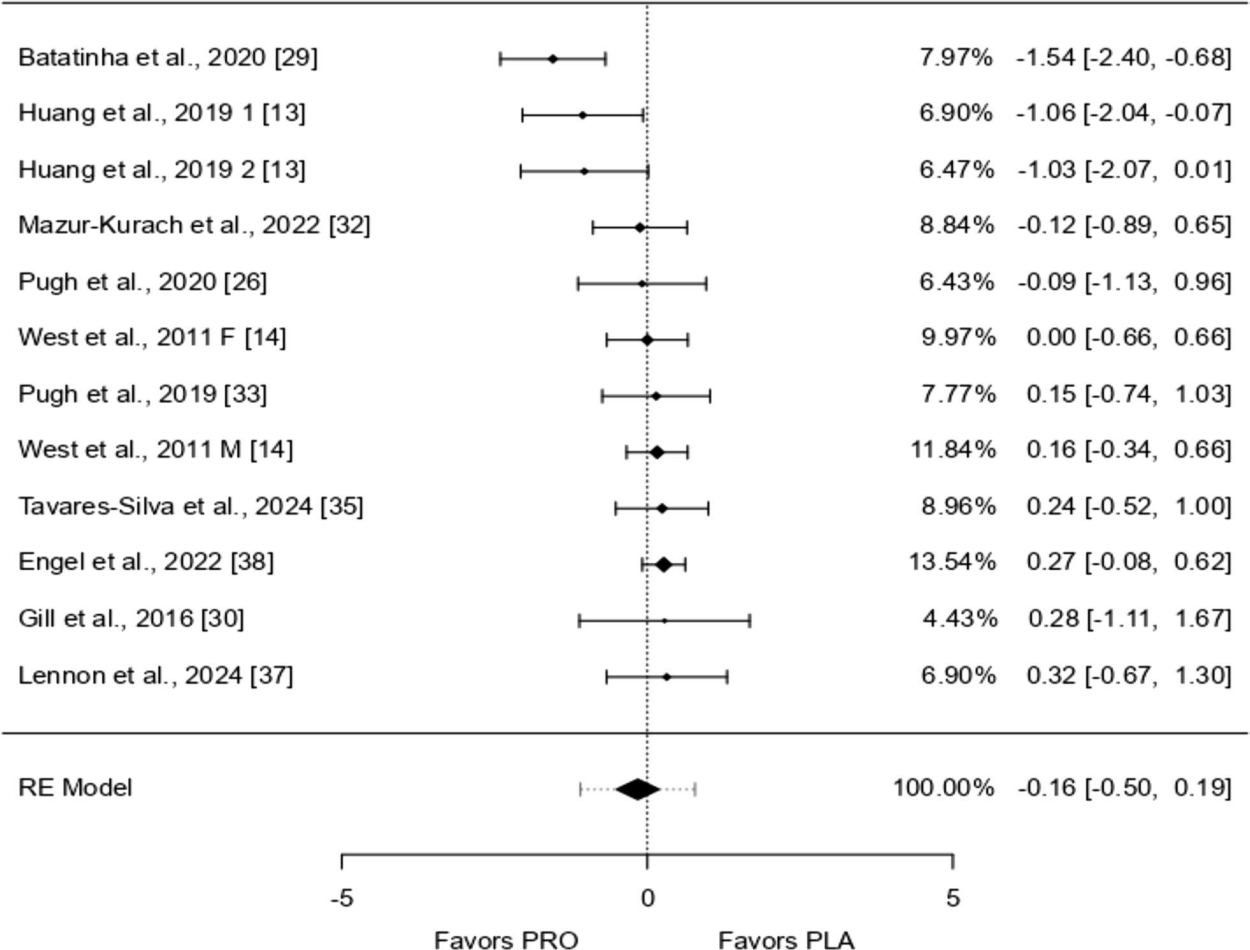
IL-8 forest plot

**Fig. 6:**
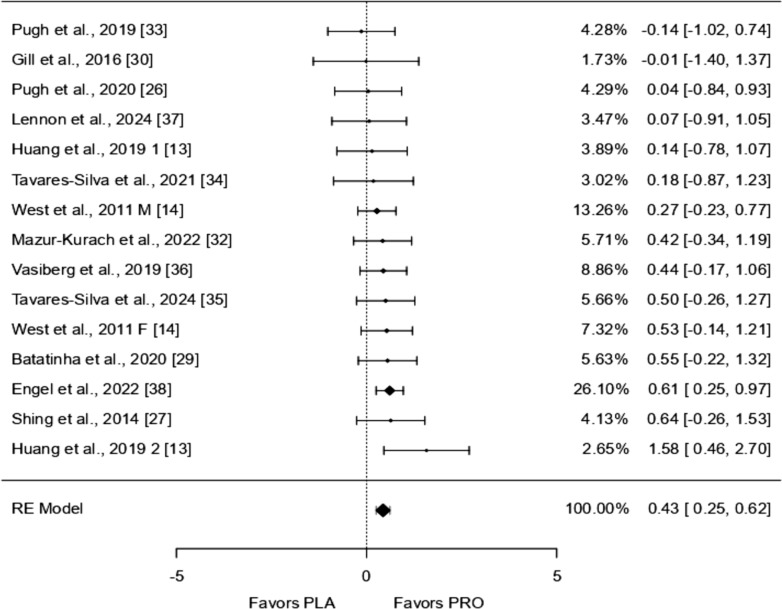
IL-10 forest plot

**Fig. 7:**
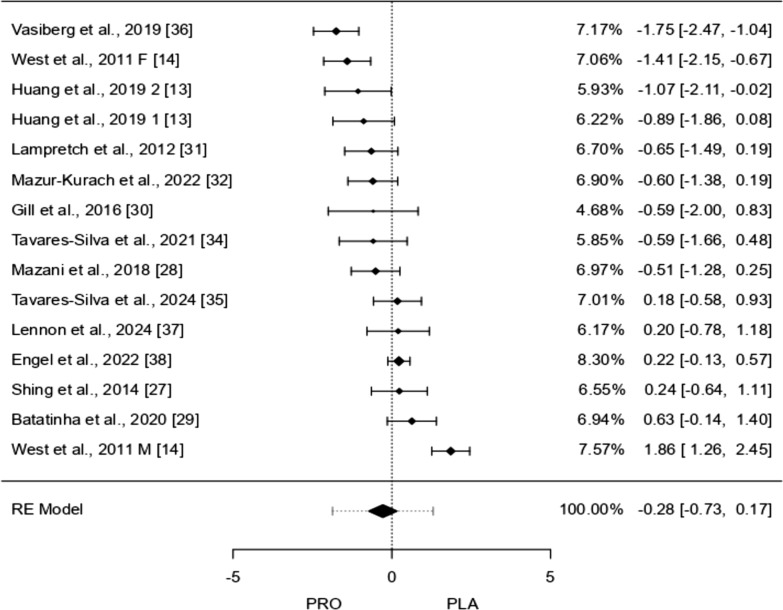
TNF-α forest plot

**Fig. 8:**
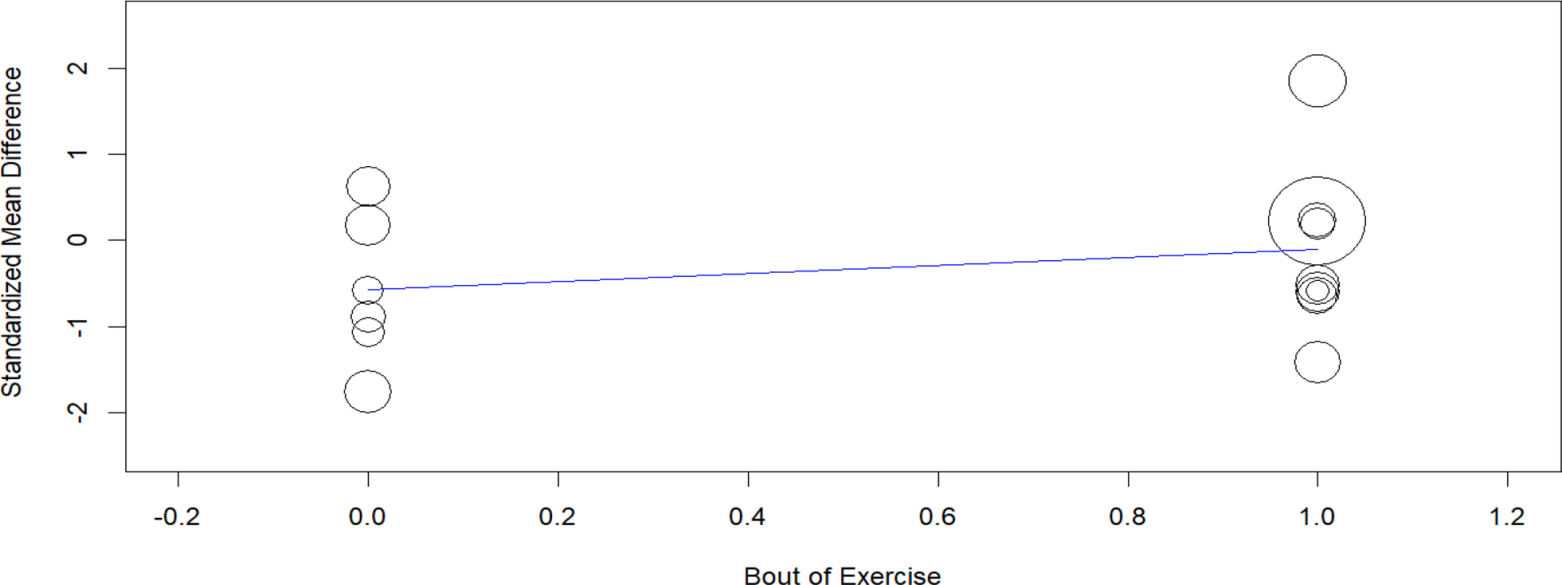
TNF-α Meta-Regression based on the bout of exercise

**Fig. 9:**
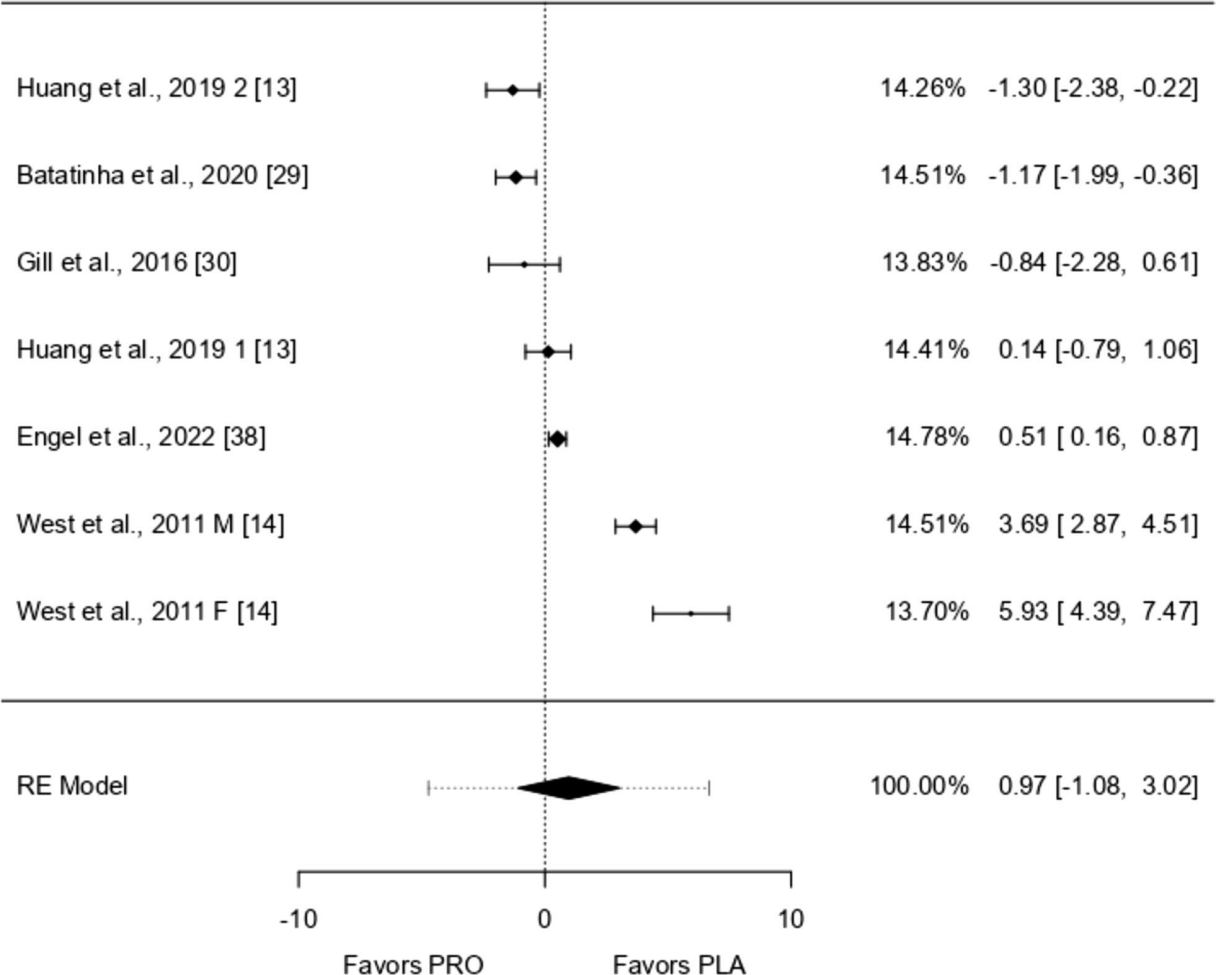
IFN-ƴ forest plot

Seven studies were analyzed, with effects ranging from − 1.527 to 0.840. Most studies (86%) showed positive effects. The overall effect, estimated using a random-effects model, was 0.065 (95% CI − 0.467 to 0.598), meaning there was no significant difference from zero (*p* = 0.810). There was considerable variation across studies (I^2^ = 73.12%, *p* = 0.0001), suggesting inconsistent findings. The prediction interval ranged from − 1.209 to 1.340, indicating that while the average effect was positive, some studies showed negative results. One study [29] appeared to be an outlier due to a large residual and high Cook’s distance, suggesting it may have strongly influenced the overall results (Figs. 3, 4, 5, 6, 7, 8 and 9).

### IL-6

Eighteen studies were analyzed, with standardized mean differences ranging from − 1.036 to 0.541. Most estimates (56%) were negative. The average standardized mean difference, calculated using a random-effects model, was − 0.124 (95% CI − 0.331 to 0.083), showing no significant difference from zero (*p* = 0.239). Although the test for heterogeneity was not significant (I^2^ = 25.11%, *p* = 0.077), some variation across studies may still be present. The prediction interval ranged from − 0.597 to 0.349, suggesting that while the average effect was negative, some studies showed positive results. No studies were identified as outliers based on studentized residuals, and Cook’s distances indicated that none had an excessive influence on the results. The regression test suggested possible funnel plot asymmetry (*p* = 0.028), but this was not confirmed by the rank correlation test (*p* = 0.081).

### IL-8

Twelve studies were included in the analysis, with standardized mean differences ranging from − 1.545 to 0.318. Most estimates (42%) were negative. The average standardized mean difference, based on a random-effects model, was − 0.156 (95% CI − 0.502 to 0.190), showing no significant difference from zero (*p* = 0.376). The Q-test indicated considerable variability between studies (I^2^ = 58.98%, *p* = 0.0113), suggesting heterogeneous outcomes. The prediction interval ranged from − 1.094 to 0.781, meaning that while the average effect was negative, some studies reported positive results. One study [29] had a high studentized residual, suggesting it might be an outlier. Additionally, its Cook’s distance indicated a potentially strong influence on the results. However, tests for publication bias did not suggest funnel plot asymmetry (*p* = 0.153 and *p* = 0.287).

### IL-10

Fifteen studies were included in the analysis, with standardized mean differences ranging from − 0.137 to 1.577. Most estimates (87%) were positive. The average standardized mean difference, based on a random-effects model, was 0.434 (95% CI 0.252 to 0.617), showing a significant effect (*p* < 0.0001). The Q-test indicated no significant variability between studies (I^2^ = 0.00%, *p* = 0.791), suggesting consistent outcomes. One study [38] had a much larger weight compared to the others, potentially influencing the results. However, no studies were identified as outliers based on studentized residuals. Cook’s distances, suggested [38] might have a strong influence on the findings. Tests for publication bias did not indicate funnel plot asymmetry (*p* = 0.623 and *p* = 0.434).

Due to its well-known anti-inflammatory profile the scale of the forest plot is inverted to mitigate the interpretation bias.

### TNF-α

Fifteen studies were analyzed, with standardized mean differences ranging from − 1.754 to 1.856. Most estimates (60%) were negative. The average standardized mean difference, based on a random-effects model, was − 0.283 (95% CI − 0.733 to 0.167), showing no significant effect (*p* = 0.218). The Q-test indicated substantial variability among studies (I^2^ = 80.57%, *p* < 0.0001), suggesting inconsistent results. The prediction interval (− 1.870–1.304) shows that while the average effect is negative, some studies reported positive outcomes. One study (West et al. [14]) had a high studentized residual and might be an outlier. Cook’s distance also suggested this study could strongly influence the results. However, tests for publication bias did not indicate funnel plot asymmetry (*p* = 0.495 and *p* = 0.208).

Table 4 presents the meta-regression statistics for TNF-α, assessing the potential influence of various covariates on its response to intervention. The intercept value (− 0.519, *p* = 0.231) suggests no significant overall effect. The duration of the intervention in weeks (− 0.004, *p* = 0.957) does not appear to significantly impact TNF-α levels, indicating that the length of the study is not a determining factor in its modulation. Similarly, the number of strains used (− 0.018, *p* = 0.815) shows no meaningful association, suggesting that variations in strain diversity do not substantially influence TNF-α changes. Although the bout of exercise (0.516, *p* = 0.331) presents a positive coefficient, it does not reach statistical significance. Moreover, the omnibus *p*-value (0.783) indicates that the overall model does not explain a significant proportion of the variance in TNF-α outcomes. These findings suggest that the examined covariates may not be strong predictors of TNF-α modulation in this meta-analysis.

**Table 4:** TNF-α meta-regression statistics

Covariate	Coefficients	Lower bound	Upper bound	Std. error	*p*-Value
Intercept	− 0.519	− 1.370	0.331	0.434	0.231
Duration in weeks	− 0.004	− 0.137	0.130	0.068	0.957
Bout of exercise	0.516	− 0.526	1.559	0.532	0.331
Number of strains	− 0.018	− 0.169	0.133	0.077	0.815
Omnibus *p*-Value			0.783		

### IFN-ƴ

Seven studies were included in the analysis, with standardized mean differences ranging from − 1.302 to 5.933. Most estimates (57%) were positive. The average standardized mean difference, based on a random-effects model, was 0.972 (95% CI − 1.077 to 3.022), which did not significantly differ from zero (*p* = 0.352). The Q-test showed considerable variability among studies (I^2^ = 97.65%, *p* < 0.0001), suggesting the results were inconsistent. The prediction interval (− 4.727–6.671) indicates that although the average outcome is positive, some studies showed negative results. One study (West et al. [14]) had a high studentized residual, potentially marking it as an outlier. However, no study appeared to have an overly strong influence on the overall results according to Cook's distances.

## Discussion

The primary objective of this meta-analysis was to evaluate the impact of probiotic supplementation on cytokine modulation following a bout of exercise in athletes. Analysis of 19 studies demonstrated that probiotic supplementation did not significantly influence most cytokine responses, including IL-1β, IL-6, IL-8, TNF-α and IFN-ƴ. However, a significant effect was noted in the upregulation of IL-10. The analysis was further stratified by exercise type, comparing VO2max tests and endurance events such as marathons and triathlons independently. Despite these distinctions, levels of IL-6, IL-8, and TNF-α remained statistically insignificant. And IL-10 remained statistically significant in all subgroup analyses. Importantly, after excluding potential outliers and highly influential studies, a significant reduction in TNF-α concentration was observed. Nevertheless, no differences were found in IL-8 levels. Additionally, TNF-α levels did not achieve statistical significance in studies focused on VO2max exercise bouts, even after accounting for outliers and highly influential studies**.**

While these results do not indicate a consistent effect across all cytokines, they suggest that probiotics may play a role in modulating immune responses and supporting recovery after exercise. Notably, the findings of this meta-analysis differ from those reported in previous meta-analyses on the same topic [9, 10]. Earlier analyses identified significant reductions in IL-6, a trend not observed in the present study. Some alignment was found regarding TNF-α modulation, yet no significant effects were reported for IL-10 in those previous meta-analyses. These discrepancies may be attributed to differences in study design, probiotic strains and dosages, exercise protocols, and participant characteristics. Additionally, a key distinction is that previous meta-analyses included studies that measured cytokine levels both at baseline and post-exercise, whereas our analysis focused on cytokine responses specifically in the context of exercise recovery. This difference in measurement timing could have influenced the observed outcomes.

It is important to highlight that both [9] and [10] included a relatively small number of studies, which may have limited the generalizability of their findings. In contrast, an umbrella review conducted by [39] examined the effects of probiotics primarily in clinical populations, such as individuals with diabetes or autoimmune diseases. In that review, no significant alignments with our results were found regarding IL-6 and TNF-α. However, another meta-analysis [40] did report a clear upregulation of IL-10, while failing to observe significant effects on IL-6 and TNF-α. This suggests that while probiotic supplementation may enhance anti-inflammatory responses in certain populations, its effects on specific cytokines can vary depending on health status, exercise intensity, probiotic formulation, and whether cytokines are measured at rest or in response to exercise.

**Fig. 10:**
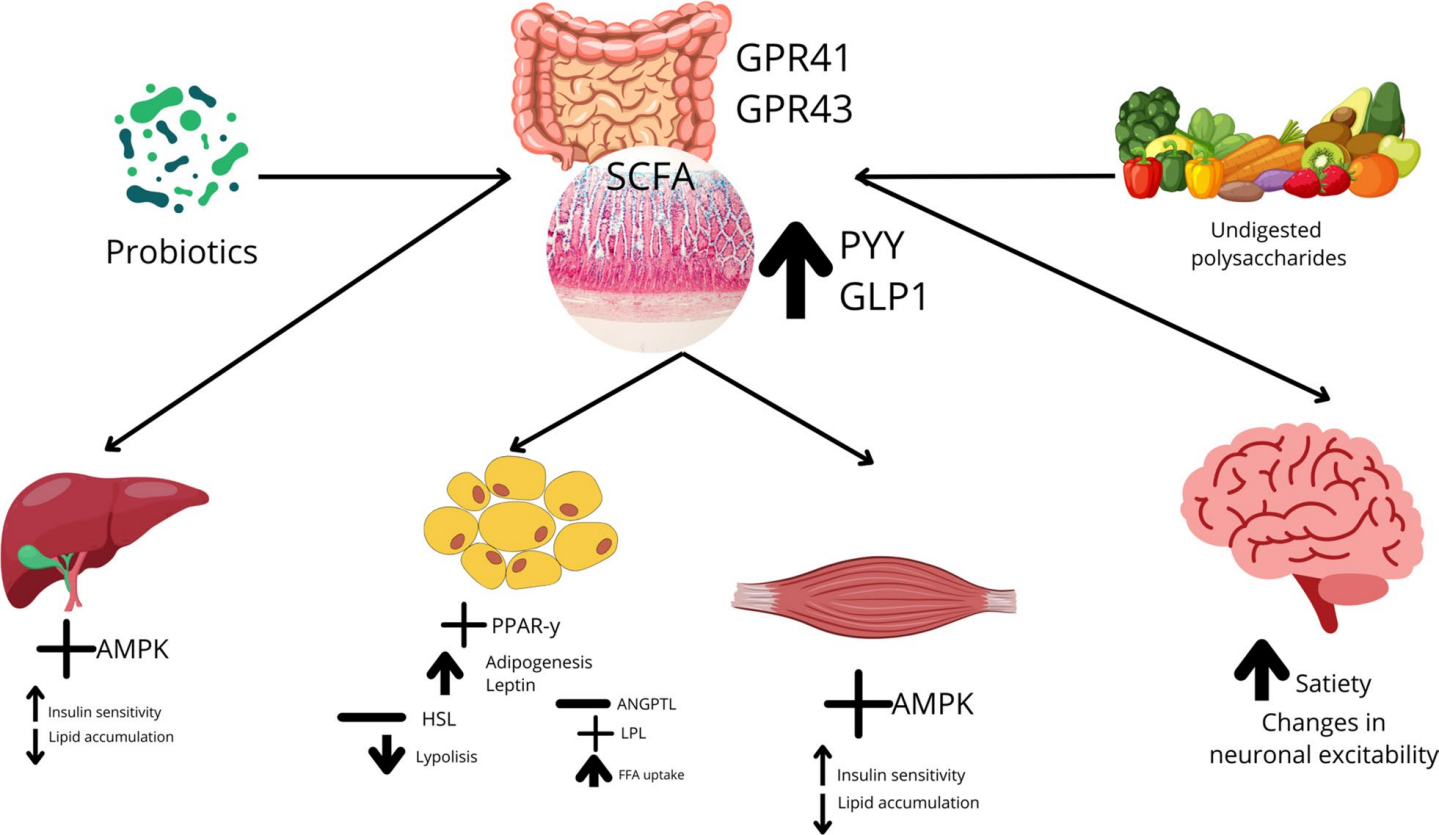
Potential Effects of Probiotics on the Modulation of SCFAs in Humans

As so, the highlighted biological mechanisms through which probiotics modulate cytokine responses are complex and diverse. Probiotics influence immune function and inflammation via several pathways: they alter the gut microbiota by increasing beneficial bacteria and decreasing harmful species, which strengthens gut barrier function and reduces systemic inflammation [2]. They also enhance the production of short-chain fatty acids (SCFAs) like butyrate, which are known for their anti-inflammatory effects. It has been shown to influence immune function and inflammation through multiple mechanisms, particularly by modulating the gut microbiota. By increasing beneficial bacterial populations and reducing the abundance of harmful species, probiotics help strengthen the gut barrier and lower systemic inflammation. They also enhance the production of short-chain fatty acids (SCFAs), such as butyrate, which have recognized anti-inflammatory effects. Notably, the impact of probiotics on SCFA production can vary depending on the specific probiotic strain used and the baseline microbial composition of the individual. Studies have indicated that probiotics such as *Lactobacillus* and *Bifidobacterium* strains can significantly increase fecal concentrations of SCFAs, including butyrate, acetate, and propionate, which are associated with improved gut health and reduced inflammation. Moreover, the production of these metabolites may also help restore microbial balance and support immune function, suggesting that individual responses to probiotics may depend on initial gut microbiota conditions [2, 6, 11]. Thus, probiotic supplementation could be a potential strategy to modulate inflammatory processes and improve immune responses, particularly in individuals with altered or compromised microbiota. The mechanism of probiotic modulation of immune function and inflammation is illustrated in Fig. 10, adapted from [11].

Figure 10 Adapted from [11] Potential effects of SCFAs in humans; AMPK: AMP kinase; ANGPTL: angiopoietin-like; GLP1: glucagon-like peptide 1; GPR: G protein-coupled receptor; HSL: hormone-sensitive lipase; LPL: lipoprotein lipase; PPAR- ƴ: peroxisome proliferator-activated receptor-ƴ; PYY: polypeptide YY

They also interact directly with immune cells, such as dendritic cells and macrophages, to boost anti-inflammatory cytokines like IL-10 while reducing pro-inflammatory cytokines such as TNF-α and IL-6 [7]. Additionally, probiotics may affect the gut-brain axis, potentially modulating stress responses and inflammation associated with exercise-induced stress and recovery [6, 11]. These combined effects contribute to reduced systemic inflammation, evident in altered cytokine profiles [1].

Getting in-depth with the results and their interpretation here are the most remarkable insights:

IL-1β is a well-known pro-inflammatory cytokine released in response to cellular damage, behaving similarly to TNF-α [7]. While estimates suggested that IL-1β concentrations were higher in the placebo group compared to the probiotic group, the clinical implications of these differences remain unclear due to the lack of statistical significance and high variability across studies. Despite this, the potential for probiotic supplementation to influence IL-1β levels warrants further exploration, particularly considering its role in modulating inflammation in response to cellular stress.

Similarly, IL-6, a cytokine with both pro-inflammatory and anti-inflammatory properties, plays a crucial role in the inflammatory response to exercise. IL-6 promotes the production of anti-inflammatory cytokines like IL-10 and IL-1Ra, which can help mitigate the harmful effects of inflammation. Probiotics may contribute to reducing systemic inflammation by enhancing gut barrier function and modulating immune cell activity [2, 7, 8]. In previous meta-analysis a reduction in the concentration of IL-6 was observed when pooling studies [9, 10, 39]. But, although estimates showed lower IL-6 concentrations in the probiotic group, the clinical relevance of this finding remains uncertain. The variability observed suggests that while probiotics may have a role in influencing IL-6, more research is needed to confirm their consistent impact on exercise-induced inflammation. However, is important to mention that the regression test for funnel plot asymmetry suggested a potential publication bias, indicating possible asymmetry in the distribution of studies. Conversely, the rank correlation test did not show a statistically significant relationship between effect sizes and study size, implying that systematic bias in effect size reporting may be less likely. These varying findings highlight the challenges in detecting publication bias, suggesting that while funnel plot asymmetry might be present, it is not consistently supported by all analyses [41].

IL-8, a cytokine involved in muscle recovery and angiogenesis, has a minimal systemic response following intense exercise, especially when eccentric movements are involved [7]. Most estimates indicated that IL-8 concentrations were lower in the probiotic group compared to the placebo group, although this difference did not reach clinical significance. Despite the variability across studies, probiotics could still have a role in moderating IL-8 levels, potentially aiding recovery by influencing localized inflammation following intense physical exertion.

**Fig. 11:**
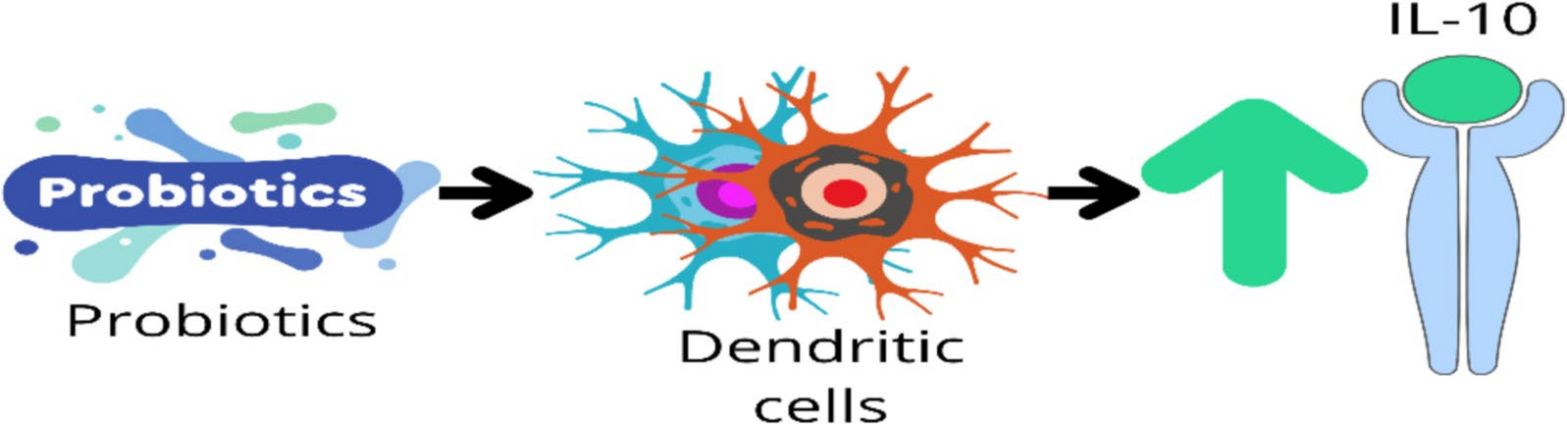
Potential Impact of Probiotics on the Upregulation of IL-10

IL-10 is widely acknowledged as a prominent anti-inflammatory cytokine, is produced by a wide range of leukocytes, IL-10 is essential for suppressing macrophage activation and the subsequent release of proinflammatory cytokines, such as IL-6 and TNF-α [42, 43]. Additionally, research has shown that probiotics can increase IL-10 concentrations by the modulation of dendritic cells [6] Fig. 11. When comparing to other meta-analysis, Compared to previous meta-analyses [9, 10], which included fewer studies and assessed cytokine levels primarily before and after exercise bouts, our analysis provides a more comprehensive evaluation. Additionally, while an umbrella review by [39] reported IL-10 upregulation, its findings were based on populations with clinical illnesses, limiting their applicability to athletes. Our meta-analysis not only demonstrates a statistically significant increase in IL-10 levels but also underscores the clinical relevance of probiotic supplementation in enhancing recovery. The observed benefits across various subgroups, including VO2max, marathon, and triathlon participants, suggest probiotics may be effective across different exercise intensities and modalities. Although some variability was noted in the subgroup analysis for marathon and triathlon participants, the overall trend of improved IL-10 levels remains clinically significant. Given IL-10’s crucial role in modulating inflammation and supporting recovery, these findings highlight probiotics as a potential strategy to optimize post-exercise recovery in athletes.

The modulation of TNF-α in response to probiotic supplementation follows a similar pattern to that of IL-1β, with evidence suggesting that probiotics can influence TNF-α levels by reducing intestinal inflammation. This effect is thought to occur through the downregulation of TLR expression and the secretion of metabolites that prevent TNF-α from entering blood mononuclear cells [11]. Despite these mechanisms, our analysis did not find a statistically significant reduction in TNF-α concentrations. Significant heterogeneity was noted across the included studies, and excluding the potential outlier study by (West et al. [14]) indicated a possible trend towards lower TNF-α levels in the probiotic group compared to placebo, although heterogeneity remained substantial. Furthermore, no single study was found to have a disproportionate influence on the overall results, and funnel plot asymmetry was not indicated by rank correlation or regression tests. It is important to note that in most studies, the prediction interval crossed zero, suggesting that previously identified effects may have been suppressed [41]. While subgroup analyses suggested potential reductions in TNF-α levels following probiotic supplementation, these findings did not reach statistical significance in either subgroup. Understanding the precise mechanisms by which probiotics may modulate TNF-α remains an area of interest, particularly in the context of inflammatory responses and recovery strategies.

The meta-regression analysis suggests that the examined covariates may not be strong predictors of TNF-α modulation in response to probiotic interventions. While neither intervention duration nor strain diversity showed a meaningful influence, exercise type emerged as a potential factor of interest. Although it did not reach statistical significance in this analysis, its role could become clearer with a larger number of studies and more standardized methodologies. Future research should focus on ensuring balanced study designs across different exercise modalities to better assess their impact on TNF-α regulation.

The modulation of IFN-γ in response to exercise remains controversial, as evidence on its regulation is inconsistent [8]. While some studies suggest that probiotic supplementation may help modulate IFN-γ levels, our analysis did not find a significant difference between groups. Higher concentrations were generally observed in the placebo group, which could indicate a heightened inflammatory response in the absence of probiotics. However, this trend was not consistent across all studies. Given IFN-γ’s role in immune regulation and inflammation, understanding its response to exercise and probiotic supplementation remains crucial for optimizing recovery strategies in athletes [44].

## Strengths and Limitations

Several factors may contribute to these discrepancies. Differences in the bouts of exercise (e.g., VO2max test vs. marathons), the design, the intensity, duration of the exercise bout, and the timing of probiotic supplementation relative to exercise are critical considerations. Additionally, the specific strains of probiotics used, their viability, and their ability to colonize the gut microbiota can significantly influence outcomes [1]. Understanding these variables is essential for interpreting the diverse results reported in the literature [41].

A notable strength of this meta-analysis is the rigorous methodology employed, including a comprehensive literature search, stringent inclusion criteria, and the use of advanced statistical techniques to address heterogeneity among studies.

The inclusion of a diverse range of studies enhances the generalizability of our findings. Moreover, the focus on cytokine modulation provides valuable insights into the immune-regulatory effects of probiotics in the context of exercise.

However, several limitations must be acknowledged. First, the potential for publication bias exists, as studies with positive findings are more likely to be published. To mitigate this, funnel plots and Egger's regression tests were employed, but some bias may still be present [41]. Regarding IL-6, potential publication bias was suggested by the Egger's regression (*p* = 0.028), indicating possible asymmetry in the funnel plot. This raises concerns about the influence of unpublished small or null-effect studies on the results, which has been acknowledged in the discussion. Heterogeneity among studies was significant, influenced by variations in study design, participant characteristics, probiotic types and dosages, and exercise protocols. To address this, analyses were conducted with at least seven studies, and subgroup analyses explored sources of heterogeneity. However, some residual heterogeneity remained, highlighting the complexity of interpreting these findings and suggesting the need for further research.

Additionally, the quality of included studies varied, with some studies having small sample sizes and short follow-up periods. The lack of standardized protocols for probiotic administration and cytokine measurement further complicates comparisons across studies. Future research should prioritize standardized methodologies to enhance the reliability and comparability of findings. Future research should focus on standardizing methodologies to improve the reliability and comparability of results. Additionally, more effective implementation of washout periods tailored to different study characteristics is recommended.

## Clinical and Practical Implications

The findings of this meta-analysis have significant clinical and practical implications for athletes and those engaged in regular physical activity. Probiotic supplementation shows promise in managing inflammation and enhancing recovery by modulating immune responses. By reducing pro-inflammatory cytokines and increasing anti-inflammatory cytokines, probiotics can mitigate exercise-induced inflammation and muscle damage, leading to faster recovery, improved performance, reduced fatigue, and a lower risk of overtraining. Additionally, probiotics may bolster immune resilience by supporting gut health, which is particularly beneficial during intense training periods [6, 11]. Based on these findings, incorporating probiotic supplementation into exercise recovery strategies could be highly beneficial. But this approach requires careful selection of probiotic strains, along with tailored dosage and timing, to meet individual needs and training schedules, effectively mitigating potential issues.

## Future Research Directions

While this meta-analysis provides valuable insights, several areas require further investigation. Long-term studies are needed to assess the sustained impact of probiotics on cytokine modulation and overall health outcomes, as some of the included studies focused on short-term effects (< 4 weeks). Additionally, future studies should consider that a minimum dosage of 1 × 10⁹ CFU, administered for at least 3 weeks, is required to achieve the aforementioned effects, as suggested by Jäger et al. [1]. Future research should also explore the role of the bout of exercise as a key factor influencing TNF-α and other cytokine modulation in response to probiotic supplementation. The type and intensity of exercise can significantly affect the inflammatory response, and understanding how these factors interact with probiotics could provide valuable insights. If future studies focus on similar populations with consistent characteristics, such as comparable baseline inflammation levels, exercise dosages, and other relevant factors, the effect of different exercise bouts (e.g., endurance versus VO₂ max tests) on TNF-α and other cytokines could be more clearly defined. This approach could advance our understanding of how probiotics influence the broader cytokine profile, particularly in terms of pro-inflammatory and anti-inflammatory cytokines, and help establish more targeted and effective protocols for probiotic supplementation based on the nature of the exercise and individual athlete profiles. Also, extending it to older adults, individuals with chronic inflammatory conditions, and different athletic populations will help understand the broader applicability of probiotics. Detailed mechanistic studies are necessary to explore the interactions between probiotics, gut microbiota, and the immune system at the molecular level. However, within this meta-analysis, only two studies (West et al. [14] and West et al. [14]) have addressed this approach.

## Conclusions

In conclusion, this meta-analysis offers strong evidence that probiotic supplementation can effectively modulate cytokine responses after exercise. By decreasing pro-inflammatory cytokines and increasing anti-inflammatory cytokines, probiotics show potential as a strategy to improve recovery and manage exercise-induced inflammation, particularly in relation to IL-10 levels. While the findings are encouraging, further research is needed to fully elucidate the long-term effects, strain-specific benefits, and mechanisms of action of probiotics in different populations. Integrating probiotics into exercise recovery protocols represents a promising avenue for improving athletic performance, reducing fatigue, and supporting immune health. Continued investigation in this field will pave the way for more personalized and effective strategies for managing exercise-induced inflammation and optimizing overall health.

## Supplementary Information

Supplementary Material 1.

Supplementary Material 2.

## Data Availability

All data and materials obtained from this research are present in this manuscript and its supplementary material and raw data can be obtained upon reasonable request.

## Abbreviations

RCTs: Randomized controlled trials
GI: Gastrointestinal
SCFAs: Short-chain fatty acids
IL: Interleukin
TNF-α: Tumor necrosis factor alpha
IL-1Ra: Interleukin 1 receptor antagonist
Th1 cells: T helper 1 cells
IFN-γ: Interferon-gamma
IgA: Immunoglobulin A
CFU: Colony-forming units
CRP: C-Reactive protein
PRISMA®: Preferred reporting items for systematic reviews and meta-analyses
WOS: Web of science
VO2max: Maximum oxygen uptake
RPM: Revolutions per minute
kJ: Kilojoules
Q-test: Cochran's Q test for heterogeneity
