# Supplementary material for: The Effect of Probiotic Supplementation on Cytokine Modulation in Athletes After a Bout of Exercise: A Systematic Review and Meta-Analysis
Source: Sports Med Open. 2025 May 22;11:58. doi: 10.1186/s40798-025-00860-7 (PMC12098250; doi:10.1186/s40798-025-00860-7)
Supplement: Supplementary file 1 — Supplementary Material 1. [file 40798_2025_860_MOESM1_ESM.docx]

**The Effect of Probiotic Supplementation On Cytokine Modulation in Athletes After a Bout of Exercise: A Systematic Review and Meta-Analysis**

Diego Aparicio Pascual^1^, Vicente Javier Clemente-Suárez^1,2^, José Francisco Tornero-Aguilera^1^, Alejandro Rubio Zarapuz^1*^

^1^Universidad Europea de Madrid. Department of Sports Sciences. Faculty of Medicine, Health and Sports, 28670 Villaviciosa de Odón, Madrid, Spain

^2^Grupo de Investigación en Cultura, Educación y Sociedad, Universidad de la Costa, Barranquilla 080002, Colombia

*Corresponding author: Alejandro Rubio Zarapuz

Alejandro.rubio@universidadeuropea.es

**Keywords:** probiotic supplementation, cytokine modulation, exercise-induced inflammation, athletes, systematic review, meta-analysis

**SUPPLEMENTARY 1 SEARCH STRING**

| Table S1 Search String | | | |
| --- | --- | --- | --- |
| Database | MeSH terms, boolean algorithms and special characters | Nº of studies | Date of search |
| Pubmed/Medline  [Title/Abstract/Keyword] | ("probiotic*" [TIAB] OR "probiotic*" [TW] OR "microbiota*" [TIAB] OR "microbiota*" [TW] OR "postbiotic*" [TIAB] OR "postbiotic*" [TW]) AND ("cytokine?" [TIAB] OR "cytokine?" [TW] OR "inflamm*" [TIAB] OR "inflamm*" [TW] OR "immune*" [TIAB] OR "immune*" [TW]) AND ("exercise" [TIAB] OR "exercise" [TW] OR "athletes" [TIAB] OR "athletes" [TW] OR "endurance athletes" [TIAB] OR "endurance athletes" [TW]) | 602 | 28^th^ of June of 2024 |
| Scopus  (Article title, Abstract, Keywrods) | ("probiotic*" OR "microbiota*" OR "postbiotic*")  AND  ("cytokine?" OR "inflamm*" OR "inmune*")  AND  ("exercise" OR "athletes" OR "endurance athletes") | 873 | 28^th^ of June of 2024 |
| Web of Science  (Topic) | ("probiotic*" OR "microbiota*" OR "postbiotic*")  AND  ("cytokine?" OR "inflamm*" OR "inmune*")  AND  ("exercise" OR "athletes" OR "endurance athletes") | 675 | 28^th^ of June of 2024 |
| Cochrane  (Title, Abstract, Keyword) | ("probiotic*" OR "microbiota*" OR "postbiotic*") AND ("cytokine?" OR "inflamm*" OR "immune*") AND ("exercise" OR "athletes" OR "endurance athletes") | 207 | 28^th^ of June of 2024 |
| Reference List (N = 1) | | | |
| Total (N = 2358) | | | |

This file contains a detailed account of all the supplementary analyses conducted.

**SUPPLEMENTARY 2 EXCLUDED ARTICLES**

Exclusion Criteria

1. Studies published prior to 2010
2. Studies where the supplementation protocol did not exclusively involved probiotics
3. Studies which did not quantify cytokines
4. Studies which quantified cytokines solely in basal state
5. Studies where participants were not athletes or were sedentary individuals
6. Studies for which full-text articles were not available

**Figure S1 Summary of Articles Excluded Based on Eligibility Criteria**

| Table S2 Excluded Articles and Its Reason | |
| --- | --- |
| Article Cite | Reason for exclusion |
| Bertuccioli et al., 2023 [1] | 3 |
| Carbuhn et al., 2018 [2] | 4 |
| Clancy et al., 2006 [3] | 1 |
| Cox et al., 2010 [4] | 4 |
| Fu et al., 2021 [5] | 3 |
| Gepner et al., 2017 [6] | 2 |
| Gill et al., 2016 [7] | 3 |
| Gleeson et al., 2011 [8] | 4 |
| Gleeson et al., 2012 [9] | 3 |
| Gleeson et al., 2016 [10] | 3 |
| Halasa et al., 2017 [11] | 3 |
| Harnett et al., 2021 [12] | 3 |
| Haywood et al., 2014 [13] | 3 |
| Herrera-Rocha et al., 2023 [14] | 2 |
| Hoffman et al., 2019 [15] | 4 |
| Huang et al., 2019 [16] | 3 |
| Huang et al., 2020 [17] | 3 |
| Ibrahim et al., 2018a [18] | 3 |
| Ibrahim et al., 2018b [19] | 5 |
| Jäger at al., 2016 [20] | 2 |
| Khani et al., 2018 [21] | 3 |
| Komano et al., 2018 [22] | 3 |
| Lee, C.-C. et al., 2022 [23] | 3 |
| Lee, M-C. et al., 2022 [24] | 3 |
| Lee, M-C. et al., 2024 [25] | 4 |
| Li et al., 2023 [26] | 3 |
| Liu, G., & Zhang, H. 2022 [27] | 3 |
| Sánchez-Macarro et al., 2021 [28] | 3 |
| Marshall et al., 2017 [29] | 3 |
| Meng et al., 2016 [30] | 4 |
| Michalickova et al., 2016 [31] | 4 |
| Michalickova et al., 2017 [32] | 3 |
| Mooren et al., 2020 [33] | 5 |
| Moreira et al., 2007 [34] | 1 |
| O’Brien et al., 2015 [35] | 4 |
| Przewlócka et al., 2023a [36] | 2 |
| Przewlócka et al., 2023b [37] | 2 |
| Pu et al., 2017 [38] | 3 |
| Pumpa et al., 2019 [39] | 3 |
| Quero et al., 2021 [40] | 2 |
| Quero et al., 2022 [41] | 2 |
| Roberts et al., 2016 [42] | 3 |
| Schreiber et al., 2021 [43] | 4 |
| Smarkusz-Zarzecka et al., 2020 [44] | 4 |
| Strasser et al., 2016 [45] | 3 |
| Townsend et al., 2018 [46] | 4 |
| Trotter et al., 2020 [47] | 3 |
| Trushina et al., 2024 [48] | 2 |
| Wang et al., 2024 [49] | 3 |
| West et al., 2012 [50] | 2 |
| Wu et al., 2024 [51] | 6 |
| Zhang et al., 2022 [52] | 2 |
| Zhang et al., 2023 [53] | 2 |

**Bibliography**

1. Bertuccioli A, Gervasi M, Annibalini G, Binato B, Perroni F, Rocchi MBL, et al. Use of Streptococcus salivarius K12 in supporting the mucosal immune function of active young subjects: A randomised double-blind study. Front Immunol. 2023 Mar 2;14:1129060; <https://www.frontiersin.org/articles/10.3389/fimmu.2023.1129060/full>
2. Carbuhn AF, Reynolds SM, Campbell CW, Bradford LA, Deckert JA, Kreutzer A, et al. Effects of Probiotic (Bifidobacterium longum 35624) Supplementation on Exercise Performance, Immune Modulation, and Cognitive Outlook in Division I Female Swimmers. Sports. 2018 Oct 10:116; <https://www.mdpi.com/2075-4663/6/4/116>
3. Clancy RL, Gleeson M, Cox A, Callister R, Dorrington M, D’Este C, et al. Reversal in fatigued athletes of a defect in interferon γ secretion after administration of *Lactobacillus acidophilus*. Br J Sports Med. 2006 Apr ;40(4):351–4; <https://bjsm.bmj.com/lookup/doi/10.1136/bjsm.2005.024364>
4. Cox AJ, Pyne DB, Saunders PU, Fricker PA. Oral administration of the probiotic *Lactobacillus fermentum* VRI-003 and mucosal immunity in endurance athletes. Br J Sports Med. 2010 Mar ;44(4):222–6; <https://bjsm.bmj.com/lookup/doi/10.1136/bjsm.2007.044628>
5. Fu SK, Tseng WC, Tseng KW, Lai CC, Tsai YC, Tai HL, et al. Effect of Daily Oral Lactobacillus plantarum PS128 on Exercise Capacity Recovery after a Half-Marathon. Nutrients. 2021 Nov 11;13(11):4023; <https://www.mdpi.com/2072-6643/13/11/4023>
6. Gepner Y, Hoffman JR, Shemesh E, Stout JR, Church DD, Varanoske AN, et al. Combined effect of *Bacillus coagulans* GBI-30, 6086 and HMB supplementation on muscle integrity and cytokine response during intense military training. Journal of Applied Physiology. 2017 Jul 1;123(1):11–8; <https://www.physiology.org/doi/10.1152/japplphysiol.01116.2016>
7. Gill SK, Teixeira AM, Rosado F, Cox M, Costa RJS. High-Dose Probiotic Supplementation Containing Lactobacillus casei for 7 Days Does Not Enhance Salivary Antimicrobial Protein Responses to Exertional Heat Stress Compared With Placebo. International Journal of Sport Nutrition and Exercise Metabolism. 2016 Apr 26(2):150–60; <https://journals.humankinetics.com/view/journals/ijsnem/26/2/article-p150.xml>
8. Gleeson M, Bishop NC, Oliveira M, Tauler P. Daily Probiotic’s (Lactobacillus casei Shirota) Reduction of Infection Incidence in Athletes. International Journal of Sport Nutrition and Exercise Metabolism. 2011 Feb ;21(1):55–64; <https://journals.humankinetics.com/view/journals/ijsnem/21/1/article-p55.xml>
9. Gleeson M, Bishop NC, Oliveira M, McCauley T, Tauler P, Lawrence C. Effects of a Lactobacillus salivarius Probiotic Intervention on Infection, Cold Symptom Duration and Severity, and Mucosal Immunity in Endurance Athletes. International Journal of Sport Nutrition and Exercise Metabolism. 2012 Aug ;22(4):235–42; <https://journals.humankinetics.com/view/journals/ijsnem/22/4/article-p235.xml>
10. Gleeson M, Bishop NC, Struszczak L. Effects of Lactobacillus casei Shirota ingestion on common cold infection and herpes virus antibodies in endurance athletes: a placebo-controlled, randomized trial. Eur J Appl Physiol. 2016 Aug ;116(8):1555–63; <http://link.springer.com/10.1007/s00421-016-3415-x>
11. Hałasa M, Maciejewska D, Baśkiewicz-Hałasa M, Machaliński B, Safranow K, Stachowska E. Oral Supplementation with Bovine Colostrum Decreases Intestinal Permeability and Stool Concentrations of Zonulin in Athletes. Nutrients. 2017 Apr 8 ;9(4):370; <https://www.mdpi.com/2072-6643/9/4/370>
12. Harnett JE, Pyne DB, McKune AJ, Penm J, Pumpa KL. Probiotic supplementation elicits favourable changes in muscle soreness and sleep quality in rugby players. Journal of Science and Medicine in Sport. 2021 Feb ;24(2):195–9; <https://linkinghub.elsevier.com/retrieve/pii/S1440244020307374>
13. Haywood BA, Black KE, Baker D, McGarvey J, Healey P, Brown RC. Probiotic supplementation reduces the duration and incidence of infections but not severity in elite rugby union players. Journal of Science and Medicine in Sport. 2014 Jul ;17(4):356–60; <https://linkinghub.elsevier.com/retrieve/pii/S1440244013001904>
14. Herrera-Rocha KM, Manjarrez-Juanes MM, Larrosa M, Barrios-Payán JA, Rocha-Guzmán NE, Macías-Salas A, et al. The Synergistic Effect of Quince Fruit and Probiotics (Lactobacillus and Bifidobacterium) on Reducing Oxidative Stress and Inflammation at the Intestinal Level and Improving Athletic Performance during Endurance Exercise. Nutrients. 2023 Nov 13 ;15(22):4764; <https://www.mdpi.com/2072-6643/15/22/4764>
15. Hoffman JR, Hoffman MW, Zelicha H, Gepner Y, Willoughby DS, Feinstein U, et al. The Effect of 2 Weeks of Inactivated Probiotic Bacillus coagulans on Endocrine, Inflammatory, and Performance Responses During Self-Defense Training in Soldiers. Journal of Strength and Conditioning Research. 2019 Sep ;33(9):2330–7; <https://journals.lww.com/10.1519/JSC.0000000000003265>
16. Huang WC, Lee MC, Lee CC, Ng KS, Hsu YJ, Tsai TY, et al. Effect of Lactobacillus plantarum TWK10 on Exercise Physiological Adaptation, Performance, and Body Composition in Healthy Humans. Nutrients. 2019 Nov 19 ;11(11):2836; <https://www.mdpi.com/2072-6643/11/11/2836>
17. Huang WC, Pan CH, Wei CC, Huang HY. Lactobacillus plantarum PS128 Improves Physiological Adaptation and Performance in Triathletes through Gut Microbiota Modulation. Nutrients. 2020 Aug 1;12(8):2315; <https://www.mdpi.com/2072-6643/12/8/2315>
18. Ibrahim NS, Ooi FK, Chen CK, Muhamad AS. Effects of probiotics supplementation and circuit training on immune responses among sedentary young males. J Sports Med Phys Fitness. 2018 Jun ;58(7–8); <https://www.minervamedica.it/index2.php?show=R40Y2018N07A1102>
19. Ibrahim NS, Muhamad AS, Ooi FK, Meor-Osman J, Chen CK. The effects of combined probiotic ingestion and circuit training on muscular strength and power and cytokine responses in young males. Appl Physiol Nutr Metab. 2018 Feb ;43(2):180–6; <http://www.nrcresearchpress.com/doi/10.1139/apnm-2017-0464>
20. Jäger R, Shields KA, Lowery RP, De Souza EO, Partl JM, Hollmer C, et al. Probiotic *Bacillus coagulans* GBI-30, 6086 reduces exercise-induced muscle damage and increases recovery. PeerJ. 2016 Jul 21 ;4:e2276; <https://peerj.com/articles/2276>
21. Khani AH, Jazayeri SMM, Ebrahimi E, Younesi-Melerdi E, Farhadi A. The *Bifidobacterim bifidum* (BIB2) Probiotic Increased Immune System Factors in Men Sprint Athletes. CNF. 2018 Jul 4 ;14(4):324–8; <http://www.eurekaselect.com/154440/article>
22. Komano Y, Shimada K, Naito H, Fukao K, Ishihara Y, Fujii T, et al. Efficacy of heat-killed *Lactococcus lactis* JCM 5805 on immunity and fatigue during consecutive high intensity exercise in male athletes: a randomized, placebo-controlled, double-blinded trial. Journal of the International Society of Sports Nutrition. 2018 Jan 5 ;15(1):39; <https://www.tandfonline.com/doi/full/10.1186/s12970-018-0244-9>
23. Lee CC, Liao YC, Lee MC, Cheng YC, Chiou SY, Lin JS, et al. Different Impacts of Heat-Killed and Viable Lactiplantibacillus plantarum TWK10 on Exercise Performance, Fatigue, Body Composition, and Gut Microbiota in Humans. Microorganisms. 2022 Nov 3 ;10(11):2181; <https://www.mdpi.com/2076-2607/10/11/2181>
24. Lee MC, Ho CS, Hsu YJ, Huang CC. Live and Heat-Killed Probiotic Lactobacillus paracasei PS23 Accelerated the Improvement and Recovery of Strength and Damage Biomarkers after Exercise-Induced Muscle Damage. Nutrients. 2022 Oct 30 ;14(21):4563; <https://www.mdpi.com/2072-6643/14/21/4563>
25. Lee MC, Hsu YJ, Chen MT, Kuo YW, Lin JH, Hsu YC, et al. Efficacy of Lactococcus lactis subsp. lactis LY-66 and Lactobacillus plantarum PL-02 in Enhancing Explosive Strength and Endurance: A Randomized, Double-Blinded Clinical Trial. Nutrients. 2024 Jun 18 ;16(12):1921; <https://www.mdpi.com/2072-6643/16/12/1921>
26. Li X, Lin Y, Chen Y, Sui H, Chen J, Li J, et al. The effects of race and probiotic supplementation on the intestinal microbiota of 10-km open-water swimmers. Heliyon. 2023 Dec ;9(12):e22735; <https://linkinghub.elsevier.com/retrieve/pii/S2405844023099437>
27. Liu G, Zhuang H. PROBIOTIC SUPPLEMENTS FOR ENDURANCE EXERCISE PERFORMANCE AND IMMUNE FUNCTION. Rev Bras Med Esporte. 2022 Apr ;28(2):99–102; <http://www.scielo.br/scielo.php?script=sci_arttext&pid=S1517-86922022000200099&tlng=en>
28. Sánchez Macarro M, Ávila-Gandía V, Pérez-Piñero S, Cánovas F, García-Muñoz AM, Abellán-Ruiz MS, et al. Antioxidant Effect of a Probiotic Product on a Model of Oxidative Stress Induced by High-Intensity and Duration Physical Exercise. Antioxidants. 2021 Feb 22 ;10(2):323; <https://www.mdpi.com/2076-3921/10/2/323>
29. Marshall H, Chrismas BCR, Suckling CA, Roberts JD, Foster J, Taylor L. Chronic probiotic supplementation with or without glutamine does not influence the eHsp72 response to a multi-day ultra-endurance exercise event. Appl Physiol Nutr Metab. 2017 Aug ;42(8):876–83; <http://www.nrcresearchpress.com/doi/10.1139/apnm-2017-0131>
30. Meng H, Lee Y, Ba Z, Peng J, Lin J, Boyer AS, et al. Consumption of *Bifidobacterium animalis* subsp. *lactis* BB‐12 impacts upper respiratory tract infection and the function of NK and T cells in healthy adults. Molecular Nutrition Food Res. 2016 May ;60(5):1161–71; <https://onlinelibrary.wiley.com/doi/10.1002/mnfr.201500665>
31. Michalickova D, Minic R, Dikic N, Andjelkovic M, Kostic-Vucicevic M, Stojmenovic T, et al. *Lactobacillus helveticus* Lafti L10 supplementation reduces respiratory infection duration in a cohort of elite athletes: a randomized, double-blind, placebo-controlled trial. Appl Physiol Nutr Metab. 2016 Jul ;41(7):782–9; <http://www.nrcresearchpress.com/doi/10.1139/apnm-2015-0541>
32. Michalickova DM, Kostic-Vucicevic MM, Vukasinovic-Vesic MDj, Stojmenovic TB, Dikic NV, Andjelkovic MS, et al. Lactobacillus helveticus Lafti L10 Supplementation Modulates Mucosal and Humoral Immunity in Elite Athletes: A Randomized, Double-Blind, Placebo-Controlled Trial. Journal of Strength and Conditioning Research. 2017 Jan ;31(1):62–70; <https://journals.lww.com/00124278-201701000-00008>
33. Mooren FC, Maleki BH, Pilat C, Ringseis R, Eder K, Teschler M, et al. Effects of Escherichia coli strain Nissle 1917 on exercise-induced disruption of gastrointestinal integrity. Eur J Appl Physiol. 2020 Jul ;120(7):1591–9; <https://link.springer.com/10.1007/s00421-020-04382-w>
34. Moreira A, Kekkonen R, Korpela R, Delgado L, Haahtela T. Allergy in marathon runners and effect of Lactobacillus GG supplementation on allergic inflammatory markers. Respiratory Medicine. 2007 Jun ;101(6):1123–31; <https://linkinghub.elsevier.com/retrieve/pii/S0954611106006020>
35. O’Brien KV, Stewart LK, Forney LA, Aryana KJ, Prinyawiwatkul W, Boeneke CA. The effects of postexercise consumption of a kefir beverage on performance and recovery during intensive endurance training. Journal of Dairy Science. 2015 Nov ;98(11):7446–9; <https://linkinghub.elsevier.com/retrieve/pii/S0022030215005834>
36. Przewłócka K, Folwarski M, Kaczmarczyk M, Skonieczna-Żydecka K, Palma J, Bytowska ZK, et al. Combined probiotics with vitamin D3 supplementation improved aerobic performance and gut microbiome composition in mixed martial arts athletes. Front Nutr. 2023 Oct 11 ;10:1256226; <https://www.frontiersin.org/articles/10.3389/fnut.2023.1256226/full>
37. Przewłócka K, Kujach S, Sawicki P, Berezka P, Bytowska ZK, Folwarski M, et al. Effects of Probiotics and Vitamin D3 Supplementation on Sports Performance Markers in Male Mixed Martial Arts Athletes: A Randomized Trial. Sports Med - Open. 2023 May 16 ;9(1):31; <https://sportsmedicine-open.springeropen.com/articles/10.1186/s40798-023-00576-6>
38. Pu F, Guo Y, He F, Zhu H, Wang S, Shen X, et al. Protective Effects of Fermented Milk with Selected Probiotics Against Acute Upper Respiratory Tract Infections by Enhancement of Immunity of the Elderly: An Open-Label Trial. The FASEB Journal. 2017 Apr 1 ;31(S1):964.25-964.25; <https://doi.org/10.1096/fasebj.31.1_supplement.964.25>
39. Pumpa KL, McKune AJ, Harnett J. A novel role of probiotics in improving host defence of elite rugby union athlete: A double blind randomised controlled trial. Journal of Science and Medicine in Sport. 2019 Aug ;22(8):876–81; <https://linkinghub.elsevier.com/retrieve/pii/S1440244018311605>
40. Quero CD, Manonelles P, Fernández M, Abellán-Aynés O, López-Plaza D, Andreu-Caravaca L, et al. Differential Health Effects on Inflammatory, Immunological and Stress Parameters in Professional Soccer Players and Sedentary Individuals after Consuming a Synbiotic. A Triple-Blinded, Randomized, Placebo-Controlled Pilot Study. Nutrients. 2021 Apr 16 ;13(4):1321; <https://www.mdpi.com/2072-6643/13/4/1321>
41. Quero-Calero CD, Abellán-Aynés O, Manonelles P, Ortega E. The Consumption of a Synbiotic Does Not Affect the Immune, Inflammatory, and Sympathovagal Parameters in Athletes and Sedentary Individuals: A Triple-Blinded, Randomized, Place-bo-Controlled Pilot Study. IJERPH. 2022 Mar 14 ;19(6):3421; <https://www.mdpi.com/1660-4601/19/6/3421>
42. Roberts J, Suckling C, Peedle G, Murphy J, Dawkins T, Roberts M. An Exploratory Investigation of Endotoxin Levels in Novice Long Distance Triathletes, and the Effects of a Multi-Strain Probiotic/Prebiotic, Antioxidant Intervention. Nutrients. 2016 Nov 17;8(11):733; <https://www.mdpi.com/2072-6643/8/11/733>
43. Schreiber C, Tamir S, Golan R, Weinstein A, Weinstein Y. The effect of probiotic supplementation on performance, inflammatory markers and gastro‐intestinal symptoms in elite road cyclists. Journal of the International Society of Sports Nutrition. 2021 Jan 2 ;18(1):36; <https://www.tandfonline.com/doi/full/10.1186/s12970-021-00432-6>
44. Smarkusz-Zarzecka J, Ostrowska L, Leszczyńska J, Orywal K, Cwalina U, Pogodziński D. Analysis of the Impact of a Multi-Strain Probiotic on Body Composition and Cardiorespiratory Fitness in Long-Distance Runners. Nutrients. 2020 Dec 7 ;12(12):3758; <https://www.mdpi.com/2072-6643/12/12/3758>
45. Strasser B, Geiger D, Schauer M, Gostner J, Gatterer H, Burtscher M, et al. Probiotic Supplements Beneficially Affect Tryptophan–Kynurenine Metabolism and Reduce the Incidence of Upper Respiratory Tract Infections in Trained Athletes: A Randomized, Double-Blinded, Placebo-Controlled Trial. Nutrients. 2016 Nov 23 ;8(11):752; <https://www.mdpi.com/2072-6643/8/11/752>
46. Townsend JR, Bender D, Vantrease WC, Sapp PA, Toy AM, Woods CA, et al. Effects of Probiotic (Bacillus subtilis DE111) Supplementation on Immune Function, Hormonal Status, and Physical Performance in Division I Baseball Players. Sports. 2018 Jul 26 ;6(3):70; <https://www.mdpi.com/2075-4663/6/3/70>
47. Trotter RE, Vazquez AR, Grubb DS, Freedman KE, Grabos LE, Jones S, et al. Bacillus subtilis DE111 intake may improve blood lipids and endothelial function in healthy adults. BM. 2020 Nov 15 ;11(7):621–30; <https://brill.com/view/journals/bm/11/7/article-p621_2.xml>
48. Trushina EN, Federal Research Centre of Nutrition, Biotechnology and Food Safety, Moscow, Mustaphina OK, Federal Research Centre of Nutrition, Biotechnology and Food Safety, Moscow, Timonin AN, Federal Research Centre of Nutrition, Biotechnology and Food Safety, Moscow, et al. Multi-strain probiotic combined with dietary fiber is an effective factor in the nutritional support of immunity in athletes. Problems of Nutrition. 2024 ;93(2):19–30; <https://www.voprosypitaniya.ru/ru/jarticles_diet/1110.html?SSr=250134d88b13ffffffff27c__07e805170d1919-264d>
49. Wang L, Meng FJ, Jin YH, Wu LQ, Tang RY, Xu KH, et al. Effects of probiotic supplementation on 12 min run performance, mood management, body composition and gut microbiota in amateur marathon runners: A double-blind controlled trial. Journal of Exercise Science & Fitness. 2024 Oct ;22(4):297–304; <https://linkinghub.elsevier.com/retrieve/pii/S1728869X2400039X>
50. West NP, Pyne DB, Cripps A, Christophersen CT, Conlon MA, Fricker PA. Gut Balance, a synbiotic supplement, increases fecal Lactobacillus paracasei but has little effect on immunity in healthy physically active individuals. Gut Microbes. 2012 May ;3(3):221–7; <http://www.tandfonline.com/doi/abs/10.4161/gmic.19579>
51. Wu SI, Lee MC, Chen WL, Huang CC. Lacticaseibacillus paracasei PS23 increases ghrelin levels and modulates microbiota composition: a post-hoc analysis of a randomized controlled study. *Food Funct.* 2024 May ;15:6523–35; <https://doi.org/10.1039/D4FO01328J>.
52. Zhang L, Liu C, Xiao H, Zhao L. Effects of probiotics supplementation on aerobic capacity and inflammatory markers in male soccer university student-athletes. *Med Sci Sports Exerc*. 2022 Sep ;54(9):487–7.
53. Zhang L, Xiao H, Zhao L, Liu Z, Chen L, Liu C. Comparison of the Effects of Prebiotics and Synbiotics Supplementation on the Immune Function of Male University Football Players. Nutrients. 2023 Feb 25 ;15(5):1158; <https://www.mdpi.com/2072-6643/15/5/1158>

**SUPPLEMENTARY 3 TABLES, FIGURES AND PLOTS NOT INCLUDED IN THE MANUSCRIPT AND SUPPLEMENTARY ANALYSIS**

1. **IL-1Β STATISTICS**

| **Table S3 Effect of IL-1β on Outcome Measures** | | | | | | | | | | | | | | | | | | | | | | | | | | | | | |
| --- | --- | --- | --- | --- | --- | --- | --- | --- | --- | --- | --- | --- | --- | --- | --- | --- | --- | --- | --- | --- | --- | --- | --- | --- | --- | --- | --- | --- | --- |
| **Random-Effects Model (k = 7)** | | | | | | | | | | | | | | | | | | | | | | | | | | | | | |
|  | | | | **Estimate** | | | | **se** | | | | **Z** | | | | | **p** | | | | **CI Lower Bound** | | | | | **CI Upper Bound** | | |  |
| Intercept | | |  | 0.0653 | | |  | 0.272 | | |  | 0.240 | | |  | | 0.810 | |  | | -0.467 | |  | | | 0.598 | |  |  |
|  | | |  |  | | |  |  | | |  |  | | |  | |  | |  | |  | |  | | |  | |  |  |
| Note. Tau² Estimator: Hedges | | | | | | | | | | | | | | | | | | | | | | | | | | | | |  |
|  | | | | | | | | | | | | | | | | | | | | | | | | | | | | |  |
| **Table S4 Heterogeneity Statistics of IL-1β** | | | | | | | | | | | | | | | | | | | | | | | | | | | | | |
| **Tau** | | **Tau²** | | | | **I²** | | | | **H²** | | | | **R²** | | | | **df** | | | | **Q** | | | **p** | | | | |
| 0.590 |  | 0.3486  (SE= 0.3241) | | |  | 73.12% | | |  | 3.720 | | |  | . | |  | | 6.000 | |  | | 27.031 | |  | < .001 | |  | | |
|  | | | | | | | | | | | | | | | | | | | | | | | | | | | | | |

1. **IL-6 STATISTICS, PUBLICATION BIAS ASSESSMENT AND FUNNEL PLOT**

| **Table S4 Effect of IL-6 on Outcome Measures** | | | | | | | | | | | | | | |
| --- | --- | --- | --- | --- | --- | --- | --- | --- | --- | --- | --- | --- | --- | --- |
| **Random-Effects Model (k = 18)** | | | | | | | | | | | | | | |
|  | | **Estimate** | | **se** | **Z** | | | | **p** | | **CI Lower Bound** | | **CI Upper Bound** | |
| Intercept |  | -0.124 |  | 0.106 | |  | -1.18 |  | 0.239 |  | -0.331 |  | 0.083 |  |
|  |  |  |  |  | |  |  |  |  |  |  |  |  |  |
| Note. Tau² Estimator: Hedges | | | | | | | | | | | | | | |
|  | | | | | | | | | | | | | | |

| **Table S5 Heterogeneity Statistics of IL-6** | | | | | | | | | | | | | | | | |
| --- | --- | --- | --- | --- | --- | --- | --- | --- | --- | --- | --- | --- | --- | --- | --- | --- |
| **Tau** | | **Tau²** | | **I²** | | **H²** | | **R²** | | **df** | | | **Q** | | **p** | |
| 0.217 |  | 0.0471  (SE= 0.0905) |  | 25.11% |  | 1.335 |  | . |  | 17.000 |  | | 25.841 |  | 0.077 |  |
|  | | | | | | | | | | | | | | | | |
|  | | | | | | | | | | | |  |  |  |  |  |
|  | | | | | | | | | | | |  |  |  |  |  |

| **Table S6 IL-6 Publication Bias Assessment** | | | | | | |
| --- | --- | --- | --- | --- | --- | --- |
| **Test Name** | | **value** | | **p** | |  |
| Fail-Safe N |  | 1.000 |  | 0.048 |  |  |
| Begg and Mazumdar Rank Correlation |  | -0.307 |  | 0.081 |  |  |
| Egger's Regression |  | -2.203 |  | 0.028 |  |  |
| Trim and Fill Number of Studies |  | 5.000 |  | . |  |  |
| Note. Fail-safe N Calculation Using the Rosenthal Approach | | | | | |  |

**Figure S2 IL-6 funnel plot**


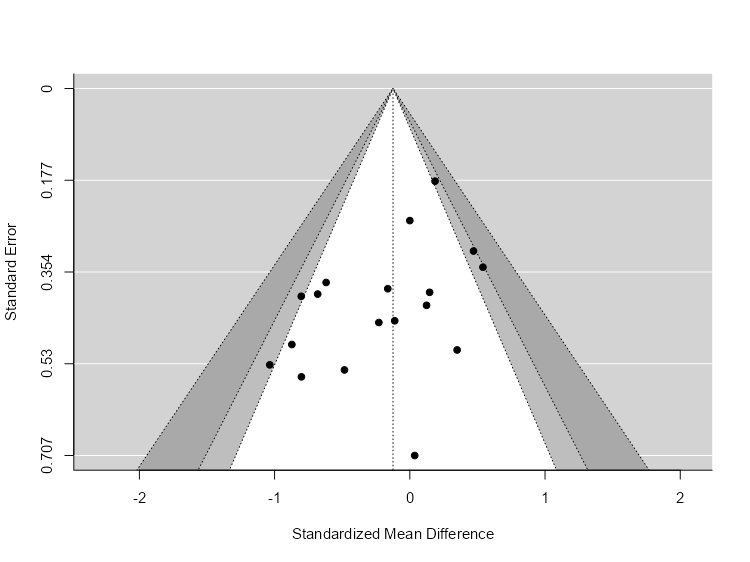


1. **IL-8 STATISTICS, PUBLICATION BIAS ASSESSMENT AND FUNNEL PLOT**

| **Table S7 Effect of IL-8 on Outcome Measures** | | | | | | | | | | | | | | |
| --- | --- | --- | --- | --- | --- | --- | --- | --- | --- | --- | --- | --- | --- | --- |
| **Random-Effects Model (k = 12)** | | | | | | | | | | | | | | |
|  | | **Estimate** | | **se** | **Z** | | | | **p** | **CI Lower Bound** | | | **CI Upper Bound** | |
| Intercept |  | -0.156 |  | 0.176 | |  | -0.885 |  | 0.376 |  | -0.502 |  | 0.190 |  |
|  |  |  |  |  | |  |  |  |  |  |  |  |  |  |
| Note. Tau² Estimator: Hedges | | | | | | | | | | | | | | |
|  | | | | | | | | | | | | | | |

| **Table S8 Heterogeneity Statistics of IL-8** | | | | | | | | | | | | | | | |
| --- | --- | --- | --- | --- | --- | --- | --- | --- | --- | --- | --- | --- | --- | --- | --- |
| **Tau** | | **Tau²** | | **I²** | | **H²** | | **R²** | | **df** | | **Q** | | **p** | |
| 0.445 |  | 0.1977  (SE= 0.1795) |  | 58.98% |  | 2.438 |  | . |  | 11.000 |  | 24.346 |  | 0.011 |  |
|  | | | | | | | | | | | | | | | |

| **Table S9 IL-8 Publication Bias Assessment** | | | | | |
| --- | --- | --- | --- | --- | --- |
| Test Name | | value | | p | |
| Fail-Safe N |  | 0.000 |  | 0.129 |  |
| Begg and Mazumdar Rank Correlation |  | -0.333 |  | 0.153 |  |
| Egger's Regression |  | -1.065 |  | 0.287 |  |
| Trim and Fill Number of Studies |  | 0.000 |  | . |  |
| Note. Fail-safe N Calculation Using the Rosenthal Approach | | | | | |
|  | | | | | |
|  | | | | | |

**Figure S3 IL-8 funnel plot**


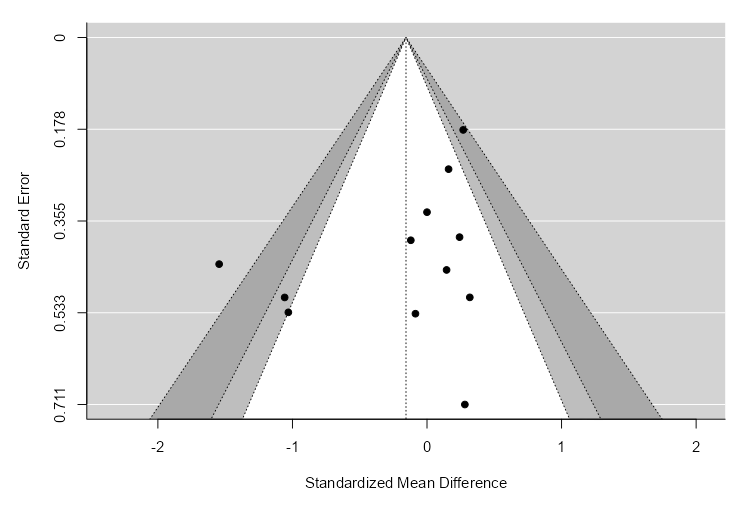


1. **IL-10 HETEROGENEITY STATISTICS, PUBLICATION BIAS ASSESSMENT AND FUNNEL PLOT**

| **Table S10 Effect of IL-10 on Outcome Measures** | | | | | | | | | | | | | |
| --- | --- | --- | --- | --- | --- | --- | --- | --- | --- | --- | --- | --- | --- |
| **Random-Effects Model (k = 15)** | | | | | | | | | | | | | |
|  | | **Estimate** | | **se** | | **Z** | | **p** | | **CI Lower Bound** | | **CI Upper Bound** | |
| Intercept |  | 0.434 |  | 0.0931 |  | 4.66 |  | < .001 |  | 0.252 |  | 0.617 |  |
|  |  |  |  |  |  |  |  |  |  |  |  |  |  |
| Note. Tau² Estimator: Hedges | | | | | | | | | | | | | |
|  | | | | | | | | | | | | | |

| **Table S11 Heterogeneity Statistics of IL-10** | | | | | | | | | | | | | | | |
| --- | --- | --- | --- | --- | --- | --- | --- | --- | --- | --- | --- | --- | --- | --- | --- |
| **Tau** | | **Tau²** | | **I²** | | **H²** | | **R²** | | **df** | | **Q** | | **p** | |
| 0.000 |  | 0 (SE= 0.0853) |  | 0% |  | 1.000 |  | . |  | 14.000 |  | 9.605 |  | 0.790 |  |
|  | | | | | | | | | | | | | | | |

| **Table S12 IL-10 Publication Bias Assessment** | | | | | |
| --- | --- | --- | --- | --- | --- |
| **Test Name** | | **value** | | **p** | |
| Fail-Safe N |  | 78.000 |  | < .001 |  |
| Begg and Mazumdar Rank Correlation |  | -0.105 |  | 0.626 |  |
| Egger's Regression |  | -0.782 |  | 0.434 |  |
| Trim and Fill Number of Studies |  | 3.000 |  | . |  |
| Note. Fail-safe N Calculation Using the Rosenthal Approach | | | | | |
|  | | | | | |
|  | | | | | |

**Figure S4 IL-10 funnel plot**


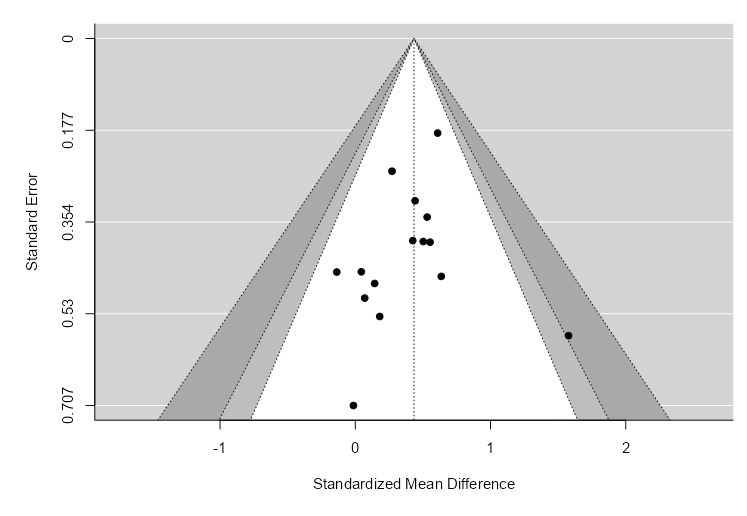


1. **TNF-Α STATISTICS, PUBLICATION BIAS ASSESSMENT, META REGRESSIONS AND FUNNEL PLOT**

| **Table S13 Effect of TNF-α on Outcome Measures** | | | | | | | | | | | | | |
| --- | --- | --- | --- | --- | --- | --- | --- | --- | --- | --- | --- | --- | --- |
| **Random-Effects Model (k = 15)** | | | | | | | | | | | | | |
|  | | **Estimate** | | **se** | **Z** | | | **p** | | **CI Lower Bound** | | **CI Upper Bound** | |
| Intercept |  | -0.283 |  | 0.229 |  | -1.23 |  | 0.218 |  | -0.733 |  | 0.167 |  |
|  |  |  |  |  |  |  |  |  |  |  |  |  |  |
| Note. Tau² Estimator: Hedges | | | | | | | | | | | | | |
|  | | | | | | | | | | | | | |

| **Table S14 Heterogeneity Statistics of TNF-α** | | | | | | | | | | | | | | | |
| --- | --- | --- | --- | --- | --- | --- | --- | --- | --- | --- | --- | --- | --- | --- | --- |
| **Tau** | | **Tau²** | | **I²** | | **H²** | | **R²** | | **df** | | **Q** | | **p** | |
| 0.776 |  | 0.6025  (SE= 0.306) |  | 80.57% |  | 5.146 |  | . |  | 14.000 |  | 93.474 |  | < .001 |  |
|  | | | | | | | | | | | | | | | |

| **Table S15 TNF-α Publication Bias Assessment** | | | | | |
| --- | --- | --- | --- | --- | --- |
| **Test Name** | | **value** | | **p** | |
| Fail-Safe N |  | 11.000 |  | 0.017 |  |
| Begg and Mazumdar Rank Correlation |  | -0.143 |  | 0.495 |  |
| Egger's Regression |  | -1.260 |  | 0.208 |  |
| Trim and Fill Number of Studies |  | 0.000 |  | . |  |
| Note. Fail-safe N Calculation Using the Rosenthal Approach | | | | | |
|  | | | | | |
|  | | | | | |

**Figure S5 TNF-α funnel plot**


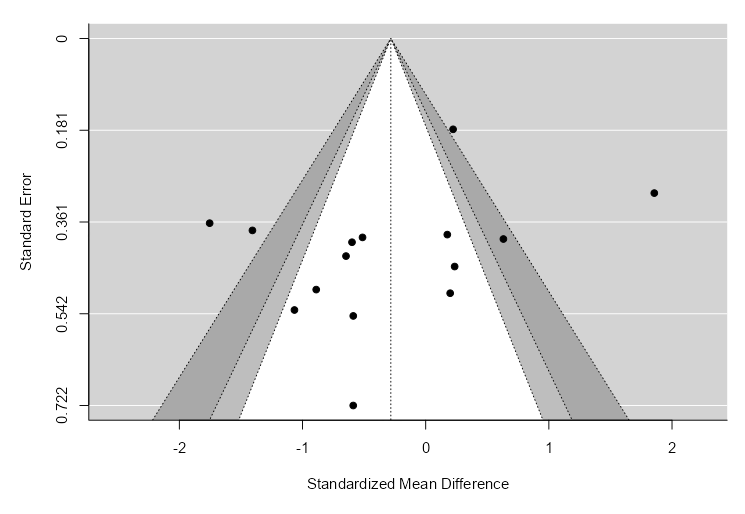


**Figure S6 TNF-α Meta-Regression based on the Duration in weeks**

**
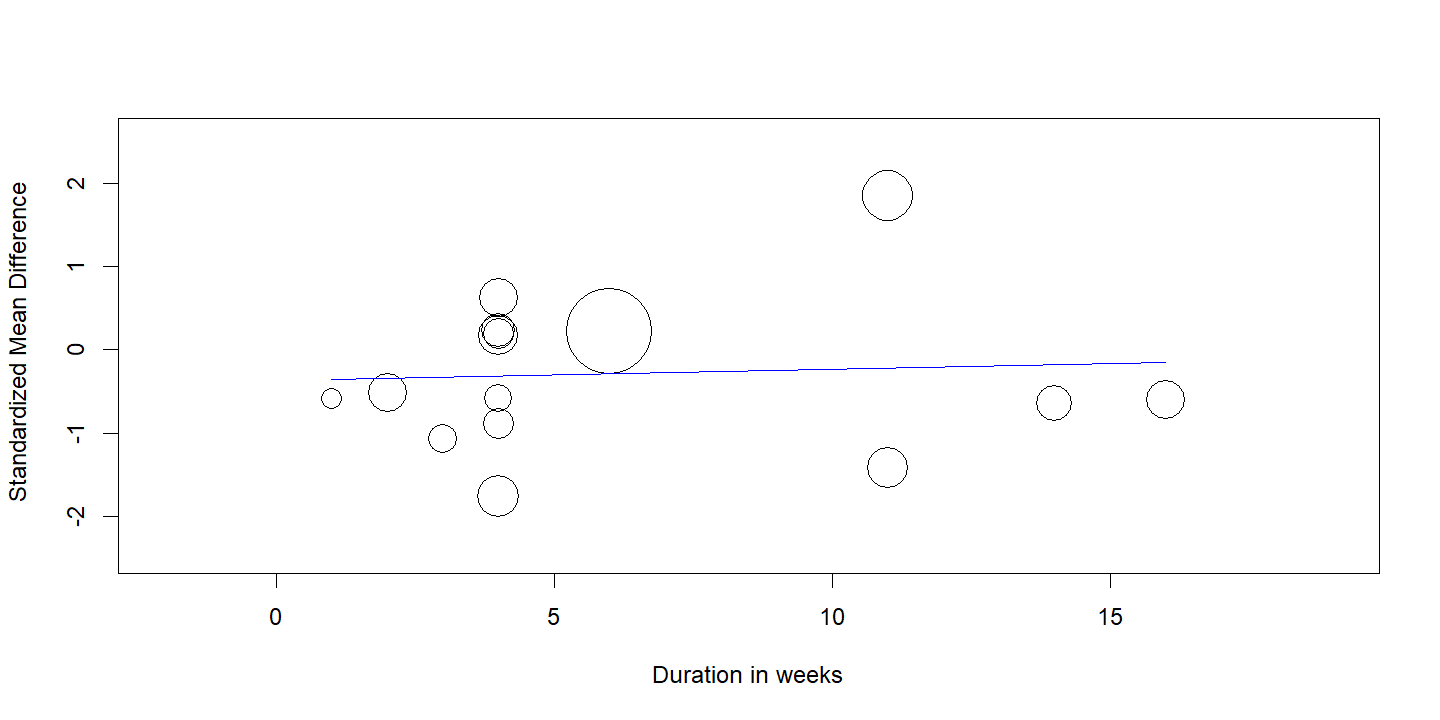
**

**Figure S7 TNF-α Meta-Regression based on the Number of strains**

**
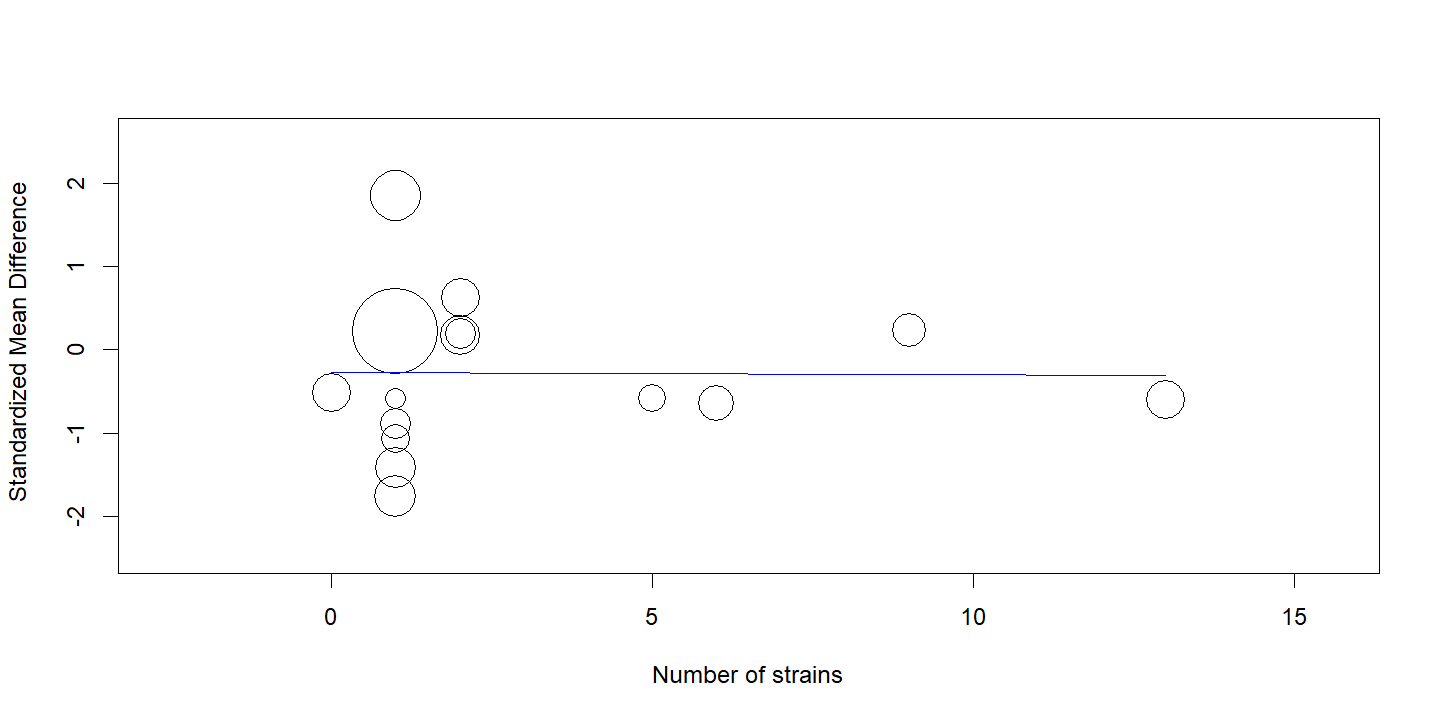
**

1. **IFN-Ƴ HETEROGENEITY STATISTICS**

| **Table S16 Effect of IFN-ƴ on Outcome Measures** | | | | | | | | | | | | | |
| --- | --- | --- | --- | --- | --- | --- | --- | --- | --- | --- | --- | --- | --- |
| **Random-Effects Model (k = 7)** | | | | | | | | | | | | | |
|  | | **Estimate** | | **se** | | **Z** | | **p** | | **CI Lower Bound** | | **CI Upper Bound** | |
| Intercept |  | 0.972 |  | 1.05 |  | 0.930 |  | 0.352 |  | -1.077 |  | 3.021 |  |
|  |  |  |  |  |  |  |  |  |  |  |  |  |  |
| Note. Tau² Estimator: Hedges | | | | | | | | | | | | | |
|  | | | | | | | | | | | | | |

| **Table S17 Heterogeneity Statistics of IFN-ƴ** | | | | | | | | | | | | | | | |
| --- | --- | --- | --- | --- | --- | --- | --- | --- | --- | --- | --- | --- | --- | --- | --- |
| **Tau** | | **Tau²** | | **I²** | | **H²** | | **R²** | | **df** | | **Q** | | **p** | |
| 2.713 |  | 7.361  (SE= 4.4216) |  | 97.65% |  | 42.487 |  | . |  | 6.000 |  | 135.649 |  | < .001 |  |
|  | | | | | | | | | | | | | | | |

**SUBGROUP ANALYSIS**

1. **IL-6 INCLUDING STUDIES REPORTING VO2max**

| **Table S18 of IL-6 including studies reporting VO2max on Outcome Measures** | | | | | | | | | | | | | |
| --- | --- | --- | --- | --- | --- | --- | --- | --- | --- | --- | --- | --- | --- |
| **Random-Effects Model (k = 11)** | | | | | | | | | | | | | |
|  | | **Estimate** | | **se** | | **Z** | | **p** | | **CI Lower Bound** | | **CI Upper Bound** | |
| Intercept |  | 0.0480 |  | 0.105 |  | 0.459 |  | 0.647 |  | -0.157 |  | 0.253 |  |
|  |  |  |  |  |  |  |  |  |  |  |  |  |  |
| Note. Tau² Estimator: Hedges | | | | | | | | | | | | | |
|  | | | | | | | | | | | | | |

| **Table S19 Heterogeneity Statistics of IL-6** | | | | | | | | | | | | | | | |
| --- | --- | --- | --- | --- | --- | --- | --- | --- | --- | --- | --- | --- | --- | --- | --- |
| **Tau** | | **Tau²** | | **I²** | | **H²** | | **R²** | | **df** | | **Q** | | **p** | |
| 0.000 |  | 0 (SE= 0.106) |  | 0% |  | 1.000 |  | . |  | 10.000 |  | 9.958 |  | 0.444 |  |
|  | | | | | | | | | | | | | | | |

A total of k=11 studies were included in the analysis. The observed standardized mean differences ranged from -0.802 to 0.541, with most estimates being positive (55%). The estimated average standardized mean difference based on the random-effects model = 0.0480 (95% CI: -0.157 to 0.253). Therefore, the average outcome did not differ significantly from zero (z = 0.459, p = 0.647). According to the Q-test, there was no significant amount of heterogeneity in the true outcomes (Q (10) = 9.958, p = 0.444, tau² = 0.0000, I² = 0.0000%). One study [38] had a relatively large weight compared to the rest of the studies (i.e., so a weight at least 3 times as large as having equal weights across studies). An examination of the studentized residuals revealed that none of the studies had a value larger than ± 2.838 and hence there was no indication of outliers in the context of this model. According to the Cook's distances, none of the studies could be overly influential. Neither the rank correlation nor the regression test indicated any funnel plot asymmetry (p = 0.165 and p = 0.198, respectively).

**Figure S8 IL-6 forest plot including studies reporting VO2max**


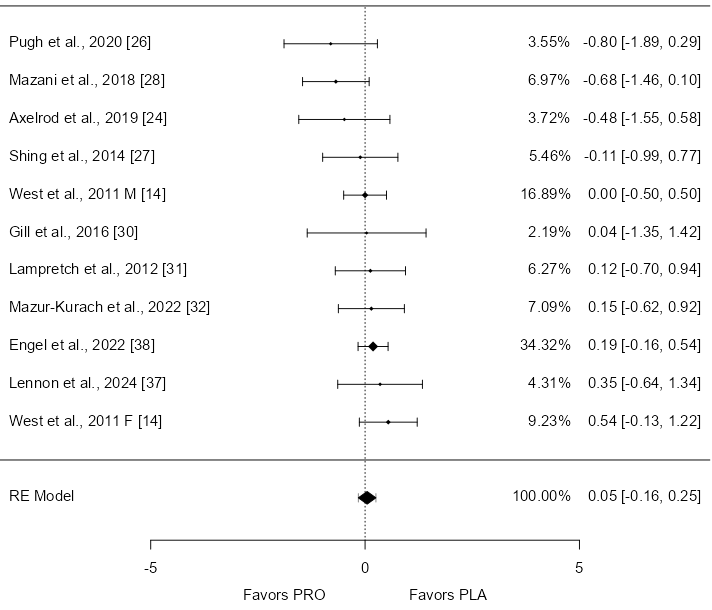


| **Table S20 IL-6 Publication Bias Assessment including studies reporting VO2max** | | | | | |
| --- | --- | --- | --- | --- | --- |
| **Test Name** | | **value** | | **p** | |
| Fail-Safe N |  | 0.000 |  | 0.467 |  |
| Begg and Mazumdar Rank Correlation |  | -0.345 |  | 0.165 |  |
| Egger's Regression |  | -1.288 |  | 0.198 |  |
| Trim and Fill Number of Studies |  | 3.000 |  | . |  |
| Note. Fail-safe N Calculation Using the Rosenthal Approach | | | | | |
|  | | | | | |

**Figure S9 IL-6 funnel plot including studies reporting VO2max**
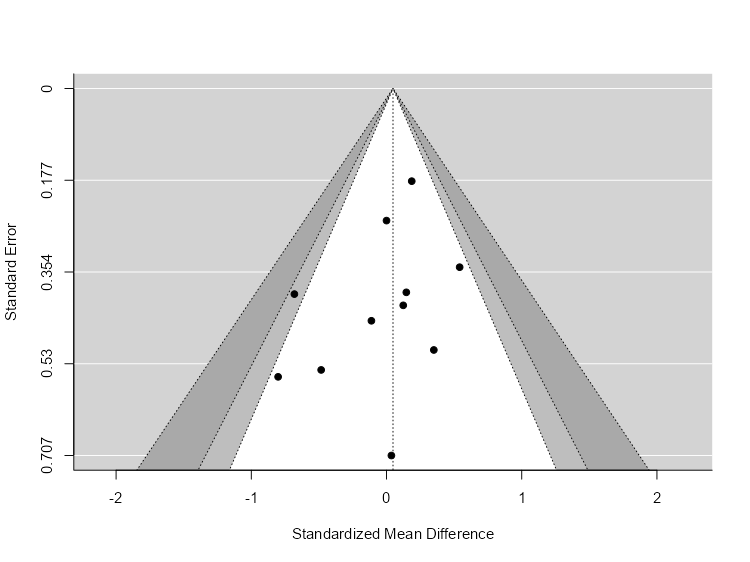


1. **IL-8 WITHOUT (BATATINHA ET AL., 2020 [29])**

| **Table S21 of IL-8 without [29] on Outcome Measures** | | | | | | | | | | | | | |
| --- | --- | --- | --- | --- | --- | --- | --- | --- | --- | --- | --- | --- | --- |
| **Random-Effects Model (k = 11)** | | | | | | | | | | | | | |
|  | | **Estimate** | | **se** | | **Z** | | **p** | | **CI Lower Bound** | | **CI Upper Bound** | |
| Intercept |  | 0.0178 |  | 0.130 |  | 0.137 |  | 0.891 |  | -0.237 |  | 0.273 |  |
|  |  |  |  |  |  |  |  |  |  |  |  |  |  |
| Note. Tau² Estimator: Hedges | | | | | | | | | | | | | |
|  | | | | | | | | | | | | | |

| **Table S22 Heterogeneity Statistics of IL-8 without [28]** | | | | | | | | | | | | | | | |
| --- | --- | --- | --- | --- | --- | --- | --- | --- | --- | --- | --- | --- | --- | --- | --- |
| **Tau** | | **Tau²** | | **I²** | | **H²** | | **R²** | | **df** | | **Q** | | **p** | |
| 0.200 |  | 0.0401  (SE= 0.1233) |  | 22.95% |  | 1.298 |  | . |  | 10.000 |  | 11.578 |  | 0.314 |  |
|  | | | | | | | | | | | | | | | |

A total of k=11 studies were included in the analysis. The observed standardized mean differences ranged from -1.058 to 0.318, with most estimates being positive (55%). The estimated average standardized mean difference based on the random-effects model was = 0.018 (95% CI: -0.237 to 0.273). Therefore, the average outcome did not differ significantly from zero (z = 0.137, p = 0.891). According to the Q-test, there was no significant amount of heterogeneity in the true outcomes (Q (10) = 11.578, p = 0.314, tau² = 0.040, I² = 22.954%). A 95% prediction interval for the true outcomes is given by -0.450 to 0.486. Hence, although the average outcome is estimated to be positive, in some studies the true outcome may in fact be negative. An examination of the studentized residuals revealed that none of the studies had a value larger than ± 2.838 and hence there was no indication of outliers in the context of this model. According to the Cook's distances, none of the studies could be overly influential. Neither the rank correlation nor the regression test indicated any funnel plot asymmetry (p = 0.218 and p = 0.177, respectively).

**Figure S10 IL-8 forest plot excluding [29]**


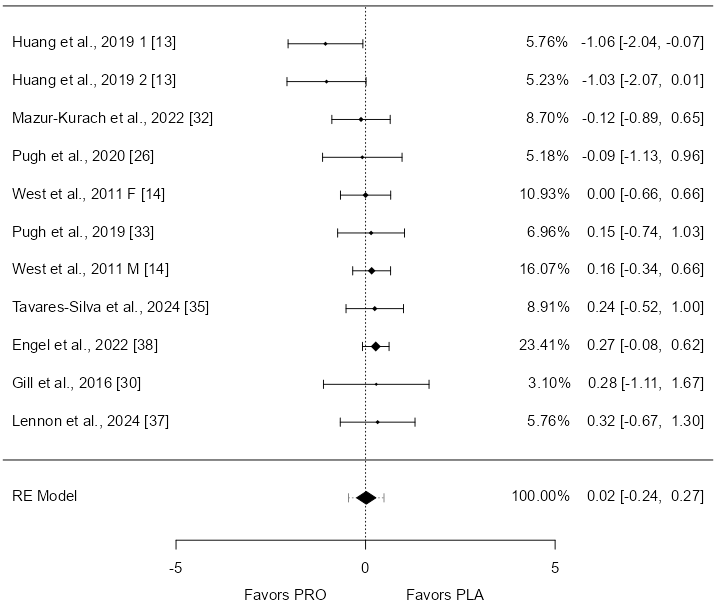


| **Table S23 IL-8 Publication Bias Assessment without [29]** | | | | | |
| --- | --- | --- | --- | --- | --- |
| **Test Name** | | **value** | | **p** | |
| Fail-Safe N |  | 0.000 |  | 0.452 |  |
| Begg and Mazumdar Rank Correlation |  | -0.309 |  | 0.218 |  |
| Egger's Regression |  | -1.351 |  | 0.177 |  |
| Trim and Fill Number of Studies |  | 0.000 |  | . |  |
| Note. Fail-safe N Calculation Using the Rosenthal Approach | | | | | |
|  | | | | | |

**Figure S11 IL-8 funnel plot excluding [29]**


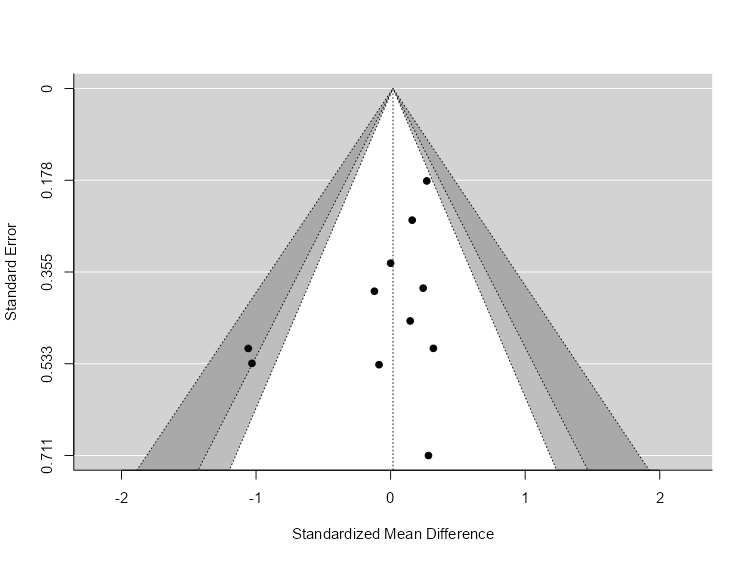


1. **IL-8 INCLUDING STUDIES REPORTING VO2max**

| **Table S24 of IL-8 including studies reporting VO2max on Outcome Measures** | | | | | | | | | | | | | |
| --- | --- | --- | --- | --- | --- | --- | --- | --- | --- | --- | --- | --- | --- |
| **Random-Effects Model (k = 7)** | | | | | | | | | | | | | |
|  | | **Estimate** | | **se** | | **Z** | | **p** | | **CI Lower Bound** | | **CI Upper Bound** | |
| Intercept |  | 0.163 |  | 0.118 |  | 1.37 |  | 0.170 |  | -0.069 |  | 0.395 |  |
|  |  |  |  |  |  |  |  |  |  |  |  |  |  |
| Note. Tau² Estimator: Hedges | | | | | | | | | | | | | |
|  | | | | | | | | | | | | | |

| **Table S25 Heterogeneity Statistics of IL-8 including VO2 max studies** | | | | | | | | | | | | | | | |
| --- | --- | --- | --- | --- | --- | --- | --- | --- | --- | --- | --- | --- | --- | --- | --- |
| **Tau** | | **Tau²** | | **I²** | | **H²** | | **R²** | | **df** | | **Q** | | **p** | |
| 0.000 |  | 0 (SE= 0.1408) |  | 0% |  | 1.000 |  | . |  | 6.000 |  | 1.442 |  | 0.963 |  |
|  | | | | | | | | | | | | | | | |

A total of k=7 studies were included in the analysis. The observed standardized mean differences ranged from -0.120 to 0.318, with most estimates being positive (57%). The estimated average standardized mean difference based on the random-effects model was = 0.163 (95% CI: -0.070 to 0.395). Therefore, the average outcome did not differ significantly from zero (z = 1.373, p = 0.170). According to the Q-test, there was no significant amount of heterogeneity in the true outcomes (Q (6) = 1.442, p = 0.963, tau² = 0.0000, I² = 0.0000%). One study [38] had a relatively large weight compared to the rest of the studies (i.e., so a weight at least 3 times as large as having equal weights across studies). An examination of the studentized residuals revealed that none of the studies had a value larger than ± 2.690 and hence there was no indication of outliers in the context of this model. According to the Cook's distances, one study [38] could be overly influential.

**Figure S12 IL-8 forest plot including studies reporting VO2max**


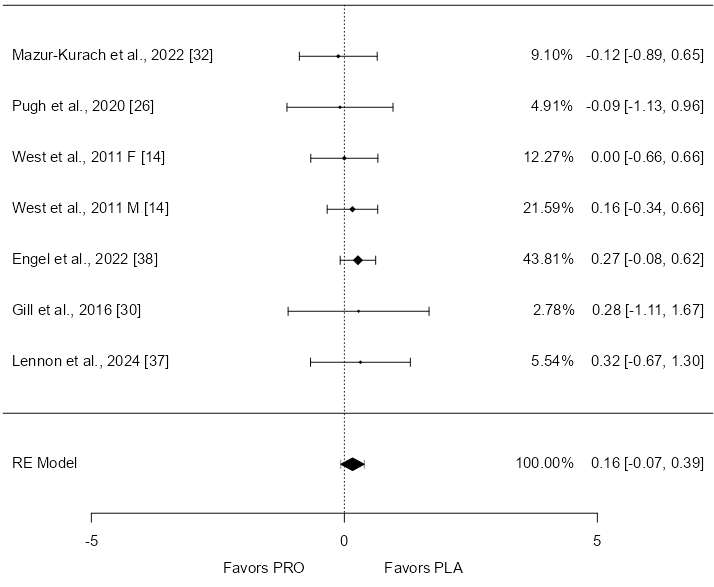


1. **IL-10 INCLUDING STUDIES REPORTING VO2max**

| **Table S26 of IL-10 including studies reporting VO2max on Outcome Measures** | | | | | | | | | | | | | |
| --- | --- | --- | --- | --- | --- | --- | --- | --- | --- | --- | --- | --- | --- |
| **Random-Effects Model (k = 8)** | | | | | | | | | | | | | |
|  | | **Estimate** | | **se** | | **Z** | | **p** | | **CI Lower Bound** | | **CI Upper Bound** | |
| Intercept |  | 0.437 |  | 0.115 |  | 3.81 |  | < .001 |  | 0.212 |  | 0.662 |  |
|  |  |  |  |  |  |  |  |  |  |  |  |  |  |
| Note. Tau² Estimator: Hedges | | | | | | | | | | | | | |
|  | | | | | | | | | | | | | |

| **Table S27 Heterogeneity Statistics of IL-10 including VO2 max studies** | | | | | | | | | | | | | | | |
| --- | --- | --- | --- | --- | --- | --- | --- | --- | --- | --- | --- | --- | --- | --- | --- |
| **Tau** | | **Tau²** | | **I²** | | **H²** | | **R²** | | **df** | | **Q** | | **p** | |
| 0.000 |  | 0 (SE= 0.1223) |  | 0% |  | 1.000 |  | . |  | 7.000 |  | 3.280 |  | 0.858 |  |
|  | | | | | | | | | | | | | | | |

A total of k=8 studies were included in the analysis. The observed standardized mean differences ranged from -0.0134 to 0.636, with most estimates being positive (88%). The estimated average standardized mean difference based on the random-effects model = 0.437 (95% CI: 0.212 to 0.662). Therefore, the average outcome differed significantly from zero (z = 3.813, p = 0.0001). According to the Q-test, there was no significant amount of heterogeneity in the true outcomes (Q (7) = 3.280, p = 0.858, tau² = 0.0000, I² = 0.0000%). One study [38] had a relatively large weight compared to the rest of the studies (i.e., so a weight at least 3 times as large as having equal weights across studies). An examination of the studentized residuals revealed that none of the studies had a value larger than ± 2.734 and hence there was no indication of outliers in the context of this model. According to the Cook's distances, one study [38] could be overly influential.

**Figure S13 IL-10 forest plot including studies reporting VO2max**

**
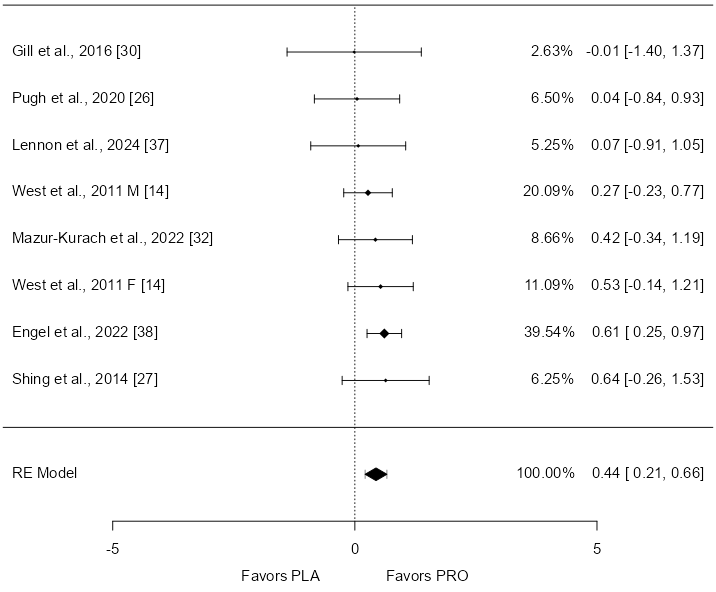
**

1. **IL-10 INCLUDING STUDIES WITH MARATHONS AND TRIATHLONS AS BOUT OF EXERCISE**

| **Table S28 of IL-10 including studies with Marathons and Triathlons as bout of exercise on Outcome Measures** | | | | | | | | | | | | | |
| --- | --- | --- | --- | --- | --- | --- | --- | --- | --- | --- | --- | --- | --- |
| **Random-Effects Model (k = 7)** | | | | | | | | | | | | | |
|  | | **Estimate** | | **se** | | **Z** | | **p** | | **CI Lower Bound** | | **CI Upper Bound** | |
| Intercept |  | 0.434 |  | 0.200 |  | 2.17 |  | 0.030 |  | 0.042 |  | 0.826 |  |
|  |  |  |  |  |  |  |  |  |  |  |  |  |  |
| Note. Tau² Estimator: Hedges | | | | | | | | | | | | | |
|  | | | | | | | | | | | | | |

| **Table S29 Heterogeneity Statistics of IL-10 including Marathons and Triathlons** | | | | | | | | | | | | | | | |
| --- | --- | --- | --- | --- | --- | --- | --- | --- | --- | --- | --- | --- | --- | --- | --- |
| Tau | | Tau² | | I² | | H² | | R² | | df | | Q | | p | |
| 0.304 |  | 0.0925  (SE= 0.177) |  | 33.52% |  | 1.504 |  | . |  | 6.000 |  | 6.323 |  | 0.388 |  |
|  | | | | | | | | | | | | | | | |

A total of k=7 studies were included in the analysis. The observed standardized mean differences ranged from -0.137 to 1.578, with most estimates being positive (86%). The estimated average standardized mean difference based on the random-effects model was = 0.434 (95% CI: 0.042 to 0.826). Therefore, the average outcome differed significantly from zero (z = 2.168, p = 0.030). According to the Q-test, there was no significant amount of heterogeneity in the true outcomes (Q (6) = 6.323, p = 0.388, tau² = 0.093, I² = 33.512%). A 95% prediction interval for the true outcomes is given by -0.280 to 1.148. Hence, although the average outcome is estimated to be positive, in some studies the true outcome may in fact be negative. An examination of the studentized residuals revealed that none of the studies had a value larger than ± 2.690 and hence there was no indication of outliers in the context of this model. According to the Cook's distances, none of the studies could be overly influential.

**Figure S14 IL-10 forest plot including studies with Marathons and Triathlons as bout of exercise**

**
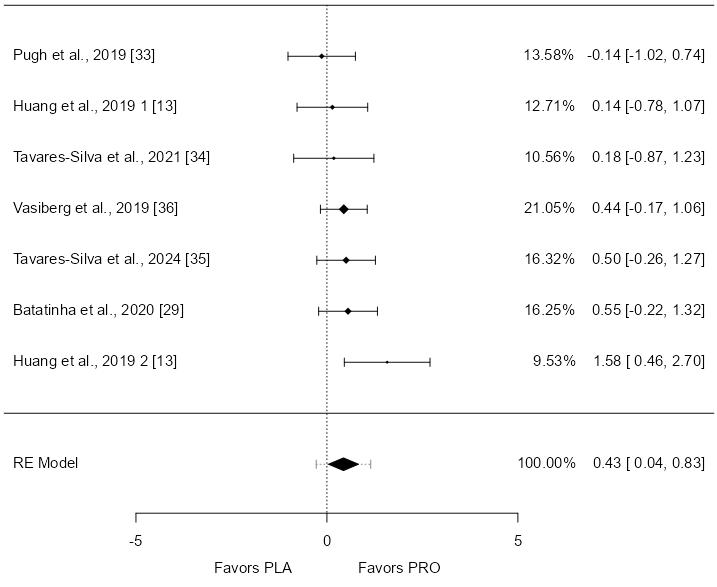
**

1. **TNF-α WITHOUT (West et al., 2011 M [14])**

| **Table S30 of TNF-α without (West et al., 2011 M [14]) on Outcome Measures** | | | | | | | | | | | | | |
| --- | --- | --- | --- | --- | --- | --- | --- | --- | --- | --- | --- | --- | --- |
| **Random-Effects Model (k = 14)** | | | | | | | | | | | | | |
|  | | **Estimate** | | **se** | | **Z** | | **p** | | **CI Lower Bound** | | **CI Upper Bound** | |
| Intercept |  | -0.447 |  | 0.180 |  | -2.48 |  | 0.013 |  | -0.800 |  | -0.094 |  |
|  |  |  |  |  |  |  |  |  |  |  |  |  |  |
| Note. Tau² Estimator: Hedges | | | | | | | | | | | | | |
|  | | | | | | | | | | | | | |

| **Table S31 Heterogeneity Statistics of TNF-α without West et al., 2011 M** | | | | | | | | | | | | | | | |
| --- | --- | --- | --- | --- | --- | --- | --- | --- | --- | --- | --- | --- | --- | --- | --- |
| **Tau** | | **Tau²** | | **I²** | | **H²** | | **R²** | | **df** | | **Q** | | **p** | |
| 0.518 |  | 0.268  (SE= 0.1912) |  | 63.73% |  | 2.757 |  | . |  | 13.000 |  | 48.047 |  | < .001 |  |
|  | | | | | | | | | | | | | | | |

A total of k=14 studies were included in the analysis. The observed standardized mean differences ranged from -1.754 to 0.631, with most estimates being negative (64%). The estimated average standardized mean difference based on the random-effects model = -0.447 (95% CI: -0.800 to -0.094). Therefore, the average outcome differed significantly from zero (z = -2.480, p = 0.013). According to the Q-test, the true outcomes appear to be heterogeneous (Q (13) = 48.047, p < 0.0001, tau² = 0.268, I² = 63.726%). A 95% prediction interval for the true outcomes is given by -1.521 to 0.627. Hence, although the average outcome is estimated to be negative, in some studies the true outcome may in fact be positive. An examination of the studentized residuals revealed that none of the studies had a value larger than ± 2.913 and hence there was no indication of outliers in the context of this model. According to the Cook's distances, none of the studies could be overly influential. Neither the rank correlation nor the regression test indicated any funnel plot asymmetry (p = 0.823 and p = 0.461, respectively).

**Figure S15 TNF-α forest plot without (West et al., 2011 M [14])**

**
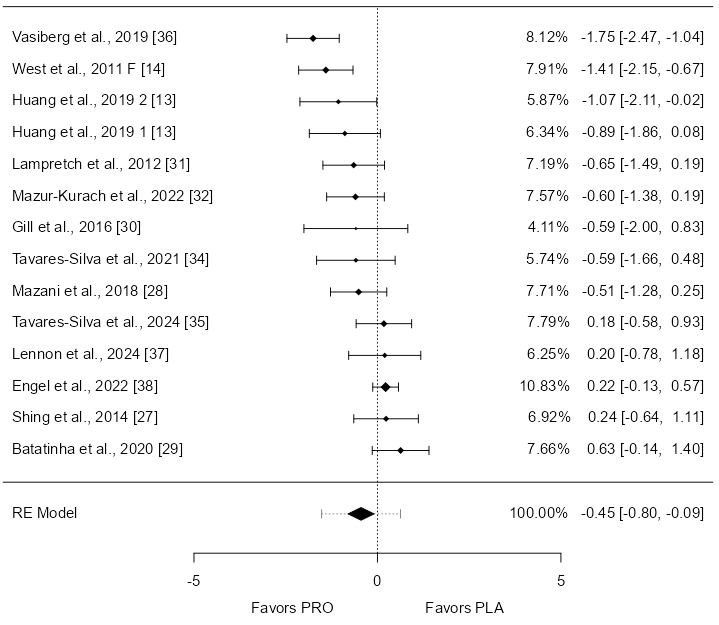
**

| **Table S32 TNF-α Publication Bias Assessment without (West et al., 2011 M [14])** | | | | | |
| --- | --- | --- | --- | --- | --- |
| Test Name | | value | | p | |
| Fail-Safe N |  | 62.000 |  | < .001 |  |
| Begg and Mazumdar Rank Correlation |  | -0.055 |  | 0.830 |  |
| Egger's Regression |  | -0.737 |  | 0.461 |  |
| Trim and Fill Number of Studies |  | 0.000 |  | . |  |
| Note. Fail-safe N Calculation Using the Rosenthal Approach | | | | | |
|  | | | | | |

**Figure S16 TNF-α funnel plot without (West et al., 2011 M [14])**

**
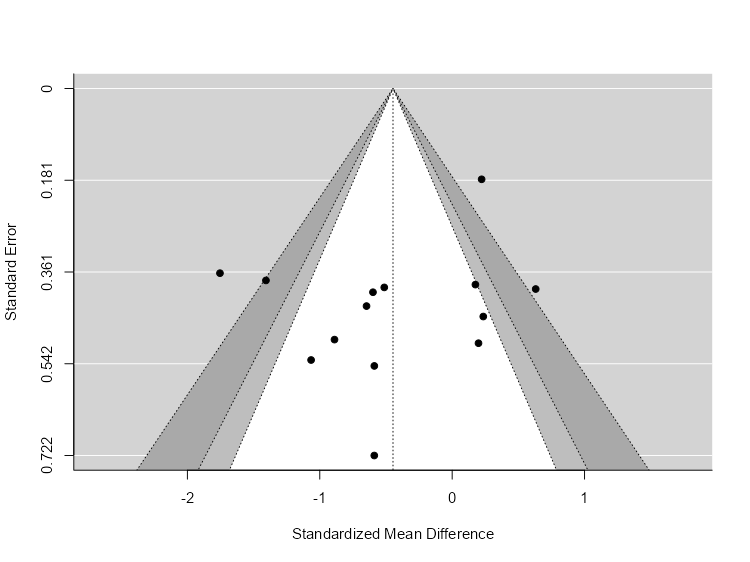
**

1. **TNF-α INCLUDING STUDIES REPORTING VO2max**

| **Table S33 of TNF-α including studies reporting VO2max on Outcome Measures** | | | | | | | | | | | | | |
| --- | --- | --- | --- | --- | --- | --- | --- | --- | --- | --- | --- | --- | --- |
| **Random-Effects Model (k = 9)** | | | | | | | | | | | | | |
|  | | **Estimate** | | **se** | | **Z** | | **p** | | **CI Lower Bound** | | **CI Upper Bound** | |
| Intercept |  | -0.102 |  | 0.304 |  | -0.335 |  | 0.738 |  | -0.697 |  | 0.494 |  |
|  |  |  |  |  |  |  |  |  |  |  |  |  |  |
| Note. Tau² Estimator: Hedges | | | | | | | | | | | | | |
|  | | | | | | | | | | | | | |

| **Table S34 Heterogeneity Statistics of TNF-αincluding VO2 max studies** | | | | | | | | | | | | | | | |
| --- | --- | --- | --- | --- | --- | --- | --- | --- | --- | --- | --- | --- | --- | --- | --- |
| **Tau** | | **Tau²** | | **I²** | | **H²** | | **R²** | | **df** | | **Q** | | **p** | |
| 0.809 |  | 0.6546  (SE= 0.4283) |  | 83.43% |  | 6.036 |  | . |  | 8.000 |  | 59.260 |  | < .001 |  |
|  | | | | | | | | | | | | | | | |

A total of k=9 studies were included in the analysis. The observed standardized mean differences ranged from -1.407 to 1.856, with most estimates being negative (56%). The estimated average standardized mean difference based on the random-effects model was = -0.102 (95% CI: -0.697 to 0.494). Therefore, the average outcome did not differ significantly from zero (z = -0.335, p = 0.738). According to the Q-test, the true outcomes appear to be heterogeneous (Q (8) = 59.260, p < 0.0001, tau² = 0.655, I² = 83.433%). A 95% prediction interval for the true outcomes is given by -1.796 to 1.592. Hence, although the average outcome is estimated to be negative, in some studies the true outcome may in fact be positive. An examination of the studentized residuals revealed that one study (West et al., 2011 M [14]) had a value larger than ± 2.773 and may be a potential outlier in the context of this model. According to the Cook's distances, two studies (West et al., 2011 F [14] & West et al., 2011 M [14]) could be overly influential.

**Figure S17 TNF-α forest plot including studies reporting VO2max**

**
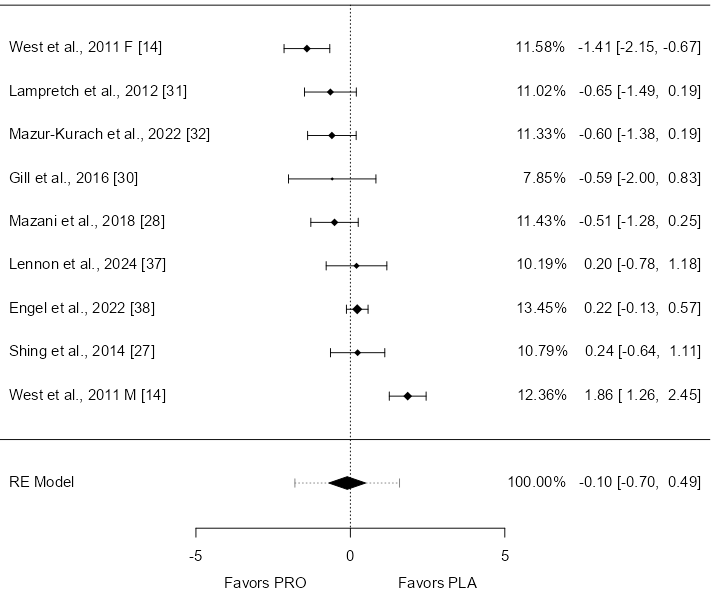
**

1. **TNF-α INCLUDING STDUIES REPORTING VO2max AND EXCLUDING (West et al., 2011 M [14])**

| **Table S35 of TNF-α including studies reporting VO2max and excluding (West et al., 2011 M [14]) on Outcome Measures** | | | | | | | | | | | | | |
| --- | --- | --- | --- | --- | --- | --- | --- | --- | --- | --- | --- | --- | --- |
| **Random-Effects Model (k = 8)** | | | | | | | | | | | | | |
|  | | **Estimate** | | **se** | | **Z** | | **p** | | **CI Lower Bound** | | **CI Upper Bound** | |
| Intercept |  | -0.344 |  | 0.190 |  | -1.81 |  | 0.070 |  | -0.716 |  | 0.028 |  |
|  |  |  |  |  |  |  |  |  |  |  |  |  |  |
| Note. Tau² Estimator: Hedges | | | | | | | | | | | | | |
|  | | | | | | | | | | | | | |

| **Table S36 Heterogeneity Statistics of TNF-α including VO2 max studies without West et al., 2011 M** | | | | | | | | | | | | | | | |
| --- | --- | --- | --- | --- | --- | --- | --- | --- | --- | --- | --- | --- | --- | --- | --- |
| **Tau** | | **Tau²** | | **I²** | | **H²** | | **R²** | | **df** | | **Q** | | **p** | |
| 0.352 |  | 0.1242  (SE=0.1882) |  | 46.91% |  | 1.884 |  | . |  | 7.000 |  | 20.361 |  | 0.005 |  |
|  | | | | | | | | | | | | | | | |

A total of k=8 studies were included in the analysis. The observed standardized mean differences ranged from -1.407 to 0.235, with most estimates being negative (62%). The estimated average standardized mean difference based on the random-effects model was = -0.344 (95% CI: -0.716 to 0.028). Therefore, the average outcome did not differ significantly from zero (z = -1.813, p = 0.070). According to the Q-test, the true outcomes appear to be heterogeneous (Q (7) = 20.361, p = 0.005, tau² = 0.124, I² = 46.912%). A 95% prediction interval for the true outcomes is given by -1.128 to 0.441. Hence, although the average outcome is estimated to be negative, in some studies the true outcome may in fact be positive. An examination of the studentized residuals revealed that one study (West et al., 2011 F [14]) had a value larger than ± 2.734 and may be a potential outlier in the context of this model. According to the Cook's distances, one study (West et al., 2011 F [14]) could be overly influential.

**Figure S18 TNF-α forest plot including studies reporting VO2max and excluding (West et al., 2011 M [14])**


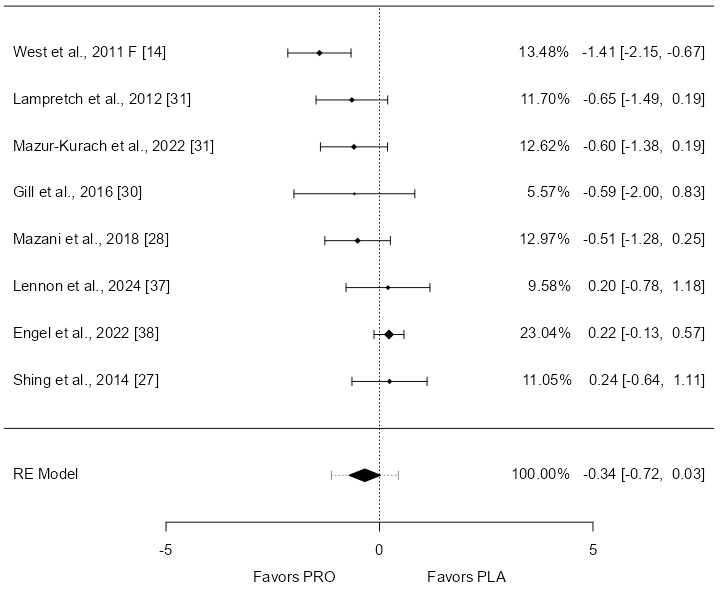


1. **TNF-α INCLUDING STUDIES REPORTING VO2max AND EXCLUDING (West et al., 2011 M [14] & West et al., 2011 F [14])**

| **Table S37 of TNF-α including studies reporting VO2max and excluding (West et al., 2011 M [14] & West et al., 2011 F [14]) on Outcome Measures** | | | | | | | | | | | | | |
| --- | --- | --- | --- | --- | --- | --- | --- | --- | --- | --- | --- | --- | --- |
| **Random-Effects Model (k = 7)** | | | | | | | | | | | | | |
|  | | **Estimate** | | **se** | | **Z** | | **p** | | **CI Lower Bound** | | **CI Upper Bound** | |
| Intercept |  | -0.0464 |  | 0.129 |  | -0.361 |  | 0.718 |  | -0.299 |  | 0.206 |  |
|  |  |  |  |  |  |  |  |  |  |  |  |  |  |
| Note. Tau² Estimator: Hedges | | | | | | | | | | | | | |
|  | | | | | | | | | | | | | |

| **Table S38 Heterogeneity Statistics of TNF-α including VO2 max studies without West et al., 2011** | | | | | | | | | | | | | | | |
| --- | --- | --- | --- | --- | --- | --- | --- | --- | --- | --- | --- | --- | --- | --- | --- |
| **Tau** | | **Tau²** | | **I²** | | **H²** | | **R²** | | **df** | | **Q** | | **p** | |
| 0.000 |  | 0  (SE=0.1442) |  | 0% |  | 1.000 |  | . |  | 6.000 |  | 8.740 |  | 0.189 |  |
|  | | | | | | | | | | | | | | | |

A total of k=7 studies were included in the analysis. The observed standardized mean differences ranged from -0.647 to 0.235, with most estimates being negative (57%). The estimated average standardized mean difference based on the random-effects model was \hat{\mu} = -0.046 (95% CI: -0.299 to 0.206). Therefore, the average outcome did not differ significantly from zero (z = -0.361, p = 0.718). According to the Q-test, there was no significant amount of heterogeneity in the true outcomes (Q (6) = 8.740, p = 0.189, tau² = 0.0000, I² = 0.0000%). One study (Engel et al., 2022 [38]) had a relatively large weight compared to the rest of the studies (i.e., so a weight at least 3 times as large as having equal weights across studies). An examination of the studentized residuals revealed that none of the studies had a value larger than ± 2.690 and hence there was no indication of outliers in the context of this model. According to the Cook's distances, one study (Engel et al., 2022 [38]) could be overly influential.

**Figure S19 TNF-α forest plot including studies reporting VO2max and excluding (West et al., 2011 M [14] & West et al., 2011 F [14])**

**
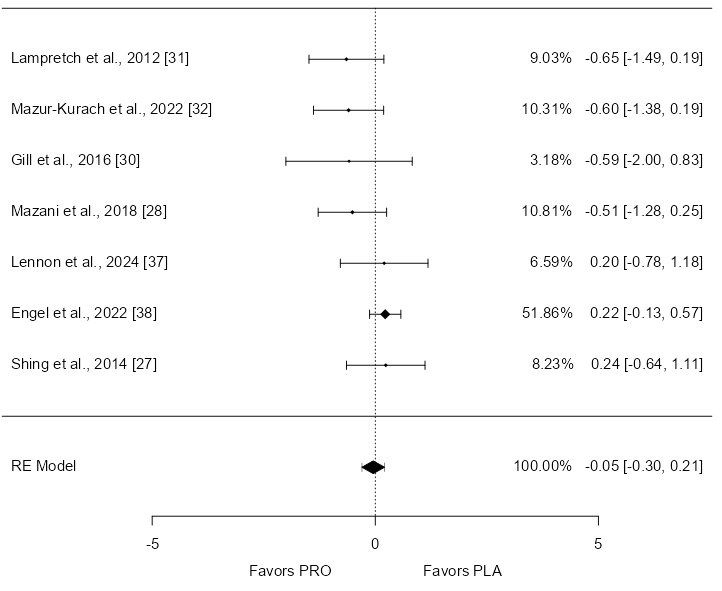
**
